# Genome Mining of Fungal Unique Trichodiene Synthase-like Sesquiterpene Synthases

**DOI:** 10.3390/jof10050350

**Published:** 2024-05-13

**Authors:** Zhanren Cong, Qiang Yin, Kunhong Tian, Njeru Joe Mukoma, Liming Ouyang, Tom Hsiang, Lixin Zhang, Lan Jiang, Xueting Liu

**Affiliations:** 1 State Key Laboratory of Bioreactor Engineering, East China University of Science of Technology, Shanghai 200237, Chinajoemukosh@gmail.com (N.J.M.);; 2 School of Environmental Sciences, University of Guelph, 50 Stone Road East, Guelph, ON N1G 2W1, Canada; 3 Department of Cardiothoracic Surgery, Children’s Hospital of Nanjing Medical University, Nanjing 210093, China

**Keywords:** fungi, genome mining, sesquiterpenes, monoterpenes, sesquiterpene synthase, trichodiene synthase

## Abstract

Sesquiterpenoids served as an important source for natural product drug discovery. Although genome mining approaches have revealed numerous novel sesquiterpenoids and biosynthetic enzymes, the comprehensive landscape of fungal sesquiterpene synthases (STSs) remains elusive. In this study, 123 previously reported fungal STSs were subjected to phylogenetic analysis, resulting in the identification of a fungi-specific STS family known as trichodiene synthase-like sesquiterpene synthases (TDTSs). Subsequently, the application of hidden Markov models allowed the discovery of 517 TDTSs from our in-house fungi genome library of over 400 sequenced genomes, and these TDTSs were defined into 79 families based on a sequence similarity network. Based on the novelty of protein sequences and the completeness of their biosynthetic gene clusters, 23 *TDTS* genes were selected for heterologous expression in *Aspergillus oryzae*. In total, 10 TDTSs were active and collectively produced 12 mono- and sesquiterpenes, resulting in the identification of the first chamipinene synthase, as well as the first fungi-derived cedrene, sabinene, and camphene synthases. Additionally, with the guidance of functionally characterized TDTSs, we found that TDTSs in Family 1 could produce bridged-cyclic sesquiterpenes, while those in Family 2 could synthesize spiro- and bridged-cyclic sesquiterpenes. Our research presents a new avenue for the genome mining of fungal sesquiterpenoids.

## 1. Introduction

Sesquiterpenoids, with over 40,000 compounds, represent one of the largest and most structurally diverse natural product families [1,2]. Their complex and diverse backbones are derived from farnesyl diphosphate (FPP) as a precursor and catalyzed by sesquiterpene synthases (STSs) [3]. These compounds exhibit a wide spectrum of bioactivities, such as antibacterial, anti-inflammatory, and cytotoxic effects, and have a wide application in the pharmaceutical industry (such as artemisinin and bilobalide), food and flavor industry (such as nootkatone and valencene), as well as biofuels (such as farnesene), with a promising economic value [4].

Fungi represent a rich source of diverse sesquiterpenes with pharmaceutical and agricultural relevance [5], including illudin M (cytotoxic), abscisic acid (plant growth regulation), and penifulvin (insecticidal) [6]. In the past decade, over 150 fungal STSs have been functionally characterized through genome mining, resulting in the discovery of numerous novel sesquiterpenes and enzymes [7]. However, the genome mining of fungal STSs has been limited to a small number of species, such as *Coprinopsis cinerea*, *Omphalotus olearius*, and *Stereum hirsutum* [7,8,9,10,11]. The sequence diversity, abundance, and distribution of fungal STSs on a global scale remain elusive. This limitation may lead to the oversight of novel sesquiterpene structures or the rediscovery of similar-function STSs.

**Figure 1:**
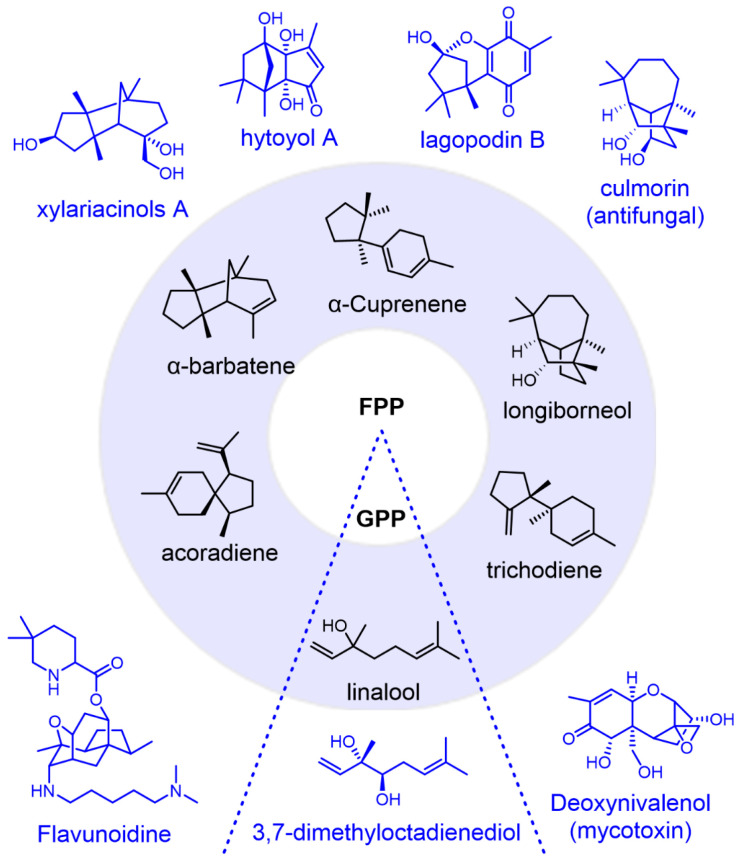
The terpene skeletons are produced by fungal TDTSs and their modified terpenoids.

Trichodiene synthase-like sesquiterpene synthase (TDTS) is a fungi-specific STS family that may catalyze the biosynthesis of complex and diverse sesquiterpenes through the bisabolene carbocation intermediate. For example, the representative trichodiene is post-modified to generate well-known trichothecene mycotoxins such as deoxynivalenol and T-2 toxin [12]. Furthermore, they can also catalyze the production of longiborneol, barbatene, and acorodiene with complex bridged or spiro structures. Two of them can catalyze the generation of monoterpenes, with only three fungal monoterpene synthases (MTSs) previously reported (Figure 1). Currently, there are only 28 reported TDTSs, the uniqueness of which suggests that further exploration may reveal more unexpected terpenoids.

Verification of the relationship between the sequences and functions of STSs could improve the discovery of new sesquiterpenes. In this study, the 123 previously reported fungal STSs were subjected to phylogenetic analysis, resulting in the identification of a fungus-specific STS family known as TDTSs. The application of hidden Markov models (HMMs) allowed the discovery of 517 TDTSs from our in-house fungi genome library. Subsequently, we constructed a sequence similarity network (SSN) to partially reveal the distribution of this special STS family, which allowed us to define all known TDTSs into 79 families based on sequence identity. Combined with the heterologous expression in *Aspergillus oryzae*, the STS responsible for the biosynthesis of chamipinene was identified for the first time, as were three key enzymes involved in the biosynthesis of cedrene, sabinene, and camphene, all characterized by fungi. Additionally, with the help of functionally characterized TDTSs, we found that TDTSs in Family 1 could produce bridged cyclic sesquiterpenes, while those in Family 2 could synthesize spiro and bridged cyclic sesquiterpenes. This study provides a foundation for the development of fungal TDTSs.

## 2. Materials and Methods

### 2.1. Strains and Media

Strains of *A. oryzae* NSAR1 (*niaD*−, *sC*−, *adeA*−, *ΔargB*) were used as the host for gene expression. The *A. oryzae* NSAR1 wildtype strain was cultivated in DPY medium for protoplast preparation (dextrose 20 g/L, polypeptone 10 g/L, yeast extract 5 g/L). *A. oryzae* NSAR1 transformants were cultivated in MA medium for transformant selection (dextrose 20 g/L, sorbitol 218.6 g/L, NH_4_Cl 2 g/L, (NH_4_)_2_SO_4_ 1 g/L, KCl 0.5 g/L, NaCl 0.5 g/L, KH_2_PO_4_ 1 g/L, MgSO_4_·7H_2_O 0.5 g/L, FeSO_4_·7H_2_O 0.02 g/L, Methionine 1.5 g/L, Adenine 0.1 g/L, agar 15 g/L, pH 5.5). *A. oryzae* transformants were also cultivated in MPY medium for fermentation (maltose 30 g/L, polypeptone 10 g/L, yeast extract 5 g/L, and (NH_4_)_2_SO_4_ 0.925 g/L). *Escherichia coli* DH10b was used for gene cloning. *E. coli* transformants were cultivated in LB medium (tryptone 10 g/L, yeast extract 5 g/L, and NaCl 10 g/L).

### 2.2. Collection and Phylogenetic Analysis of Reported Fungal STSs

A total of 123 reported fungal STS sequences were collected from published papers and public databases (Genebank [http://www.ncbi.nlm.nih.gov/ (accessed on 8 December 2023)], UniProtKB [http://www.uniprot.org/ (accessed on 08/12/2023)], and JGI [http://www.jgi.doe.gov/ (accessed on 8 December 2023)]). The accession number, gene name, organism, products, and cyclization pathway of all reported STSs are summarized in Tables S1 and S2. Fungal STSs were labeled as germacrene cyclization intermediate derived, humulene cyclization intermediate derived, or bisabolene cyclization intermediate derived according to their products and cyclization intermediates. The cyclization intermediates for each sesquiterpene were determined using the scheme detailed in IUBMB’s Enzyme Nomenclature Supplement 24 (2018) [13]. The cyclization intermediates for STSs with multiple products were determined by their major products. The sequence alignment of 123 previously reported TDTSs was made in Clustal Omega 1.2.2 with default parameters [14]. Phylogenetic analysis of fungal STSs was performed in MEGA7 [15] using the maximum-likelihood method with the Jones–Taylor–Thornton (JTT) model, and default parameters were used. The phylogenetic tree was rendered using iTOL 6.9 [16].

### 2.3. Extraction and SSN Analysis of TDTSs from Fungi Genome Database

A profile hidden Markov model (pHMM) was built with default parameters using 27 reported TDTSs to screen TDTSs from the in-house fungal genome database (>450 genomes) with the default parameters (Table S1), resulting in the extraction of a total of 1513 sequences [17]. To exclude the STSs belonging to the terpene synthase (TPTS) family from these sequences, we built another pHMM using 98 reported TPSs (Table S2). Each sequence extracted from the in-house genome database was scored by two HMM profiles using HMMer 3.0. The 965 TDTS sequences were selected with higher scores on TDTS pHMM. These 965 sequences were further de-replicated by CD-Hit v4.8.1 with a sequence identity cut-off set at 0.8, resulting in 517 TDTS sequences. TDTSs are then located in their respective genomes using BLAST [18], followed by the extraction of the biosynthetic gene clusters (BGCs) within 10 kb upstream and downstream of the identified TDTSs. The genes on BGCs were predicted by AUGUSTUS 3.5.0 [19], with *Aspergillus oryzae* as the reference organism. The sequence identity of these predicted TDTSs, together with the reported ones, was calculated using BLAST 2.2.30 to generate SSN profiles with default parameters. SSNs were then visualized using Cytoscape 3.6.0 with an e-value cut-off set at 10^−75^ [20]. The alignment of all selected TDTSs and reported TDTSs was completed using Clustal Omega 1.2.2 with default parameters and visualized with Jalview 2.11.3.2 [21]. The visualization of the HMM logos of TDTS and TPTS pHMM was finished using Skyline [22] with default parameters.

### 2.4. Construction of Expression Plasmids

The 8 TDTS genes were synthesized by GenScript Biotech (Shanghai, China). The other 15 TDTS genes were amplified from the gDNA of corresponding wild strains with primers shown in Tables S3 and S4. PCR reactions were performed with the Q5^®^ High-Fidelity DNA Polymerase (NEB biotech, Ipswich, MA, USA). pUARA4 was digested utilizing *Kpn*I (NEB biotech). The ClonExpress^®^II One Step Cloning Kit (Vazyme Biotech, Nanjing, China) was used to construct expression plasmids by inserting the PCR product of different TDTS genes into the *Kpn*I restriction sites of pUARA4 through homologous recombination to produce heterologous expression plasmids, pUARA4-TDTS. The single clones were manually picked out and utilized as the template for PCR using 2 × Taq Plus Master Mix (Vazyme) with the primers listed in Table S4. Positive clones were sequenced by Tsingke Biotech to confirm the gene was inserted into the right restriction site with no mutations. Plasmid DNA was extracted using the Plasmid Mini Kit I (OMEGA, Norcross, GA, USA). The active *TDTS* sequences were deposited in the National Center for Biotechnology Information under accession number PP516626-35. The 13 inactive *TDTS* sequences deposited in the National Center for Biotechnology Information under accession number PP776614-26

### 2.5. Preparation of Protoplasts and Transformation of A. oryzae

*A. oryzae* was selected as the heterologous expression host, and the protoplast–polyethylene glycol method was used for the transformant’s construction. Spores of *A. oryzae* NSAR1 (1.0 × 10^8^ cells) were placed into DPY medium (50 mL) in a 250 mL flask and shaken at 220 rpm at 30 °C for 2 days. Hyphae were collected through a filter, followed by washing with a 0.8 M NaCl solution 2–3 times and drying. We then dissolve 100 mg of Yatalase (TAKARA) in 20 mL of Solution 0 (0.8 M NaCl, 10 mM KH_2_PO_4_) and filter the solvent to sterilize. We then dissolved 100 mg of yatalase (TAKARA, Kusatsushi, Japan) in 20 mL of Solution 0 (0.8 M NaCl, 10 mM KH_2_PO_4_), and the liquid was filtered through a 0.22 μm pore size filter to sterilize. Then, the hyphae were placed in Yatalase solution and incubated at 30 °C and 220 rpm for 2–3 h until most of the hyphae disintegrated. The protoplasts were collected by filtering and washing with miracloth (Merck, Rahway, NJ, USA) and washed with a 0.8 M NaCl solution three times. Finally, the concentration of protoplasts was adjusted to 1.0 × 10^8^ cells/mL with solution 2 (Sorbitol 218.6 g/L, CaCl_2_·2H_2_O 7.35 g/L, NaCl 2.05 g/L, 1 M Tris-HCl 10 mL/L). Then, 200 μL prepared protoplasts and 10 μg heterologous expression plasmid were mixed, followed by incubating on ice for 20 min. Then 1 mL Solution 3 (PEG 600 g/L, CaCl_2_·2H_2_O 7.35 g/L, 1 M Tris-HCl 10 mL/L) was added, followed by incubation at room temperature for 20 min. Then, 10 mL Solution 2 was added, followed by centrifugation at 4 °C at 800× *g* for 10 min. The supernatant was discarded, and the precipitation was resuspended in 1 mL of Solution 2. The supernatant was spread onto MA medium and cultured at 30 °C for 3–5 days. After 3–5 days, single colonies were picked and transferred to new MA medium plates. After 2–3 rounds of transfer, these AO (*A. oryzae*) transformants were verified by picking up single colonies manually and suspending them in 100 μL NaOH solution (25 mM) and lysed through heating at 100 °C for 10 min. The supernatant was used as the template for PCR using 2 × Taq Plus Master Mix (Vazyme) with the primers listed in Table S4. At least three positive transformants were selected for fermentation and metabolite analysis. A series of AO-*TDTS* transformants (harboring *TDTS*) were constructed utilizing this method [10]; details are summarized in Table S5.

### 2.6. Fermentation and Analysis of the Metabolites from A. oryzae Transformants

*A. oryzae* transformants were inoculated into MPY medium (5 mL) in a 20 mL headspace screw-top vial and incubated at 30 °C and 220 rpm for 3 days. The volatile compounds produced by *A. oryzae* transformants were extracted with a solid-phase microextraction (SPME) fiber (50/30 μm DVB/CAR/PDMS; Stableflex, 24Ga, manual holder) for 15 min at room temperature. After extraction, the SPME fiber was inserted into the injection port of a QP2010SE (Shimadzu, Kyoto, Japan) gas chromatography–mass spectrometry (GC–MS) apparatus with a DB5 MS capillary column (0.25 mm × 30 m, 0.25 μm film thickness, SHIMADZU) for 10 s. The SPME fiber was maintained in the injection port to avoid contamination. The temperature of the injection port was set at 250 °C, and the GC–MS program was started in splitless mode. The column temperature was increased at 30 °C/min from 60 °C to 120 °C, increased at 5 °C/min to 180 °C, then increased at 30 °C/min to 270 °C. The flow rate of the helium carrier gas was 1.41 mL/min. The *m*/*z* detection range was set at 50–250. The structures of compounds detected by GC–MS were identified by comparing their mass spectra with data on spectra of terpenoids at the National Institute of Standards and Technology (NIST 14) standard reference database.

## 3. Results

### 3.1. Identification of TDTSs through Phylogenetic Analysis of Fungal STSs

**Figure 2:**
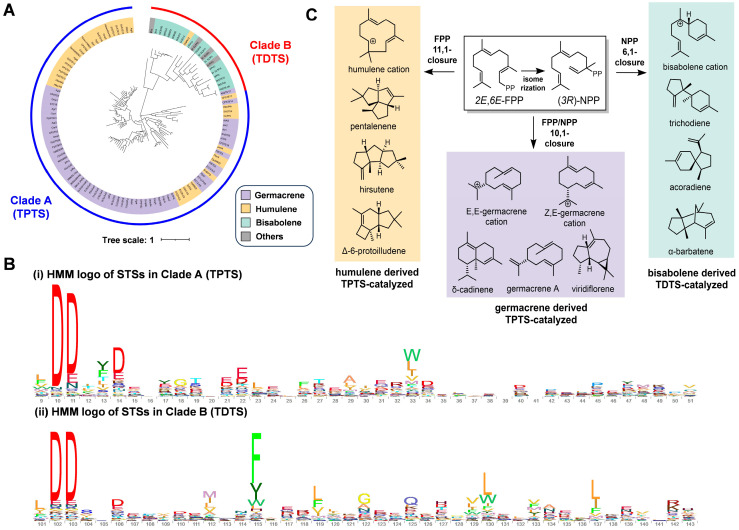
The phylogenetic analysis of reported fungal STSs revealed a fungi-specific TDTS family. (**A**) The phylogenetic analysis of fungal STSs classified them into two families: TPTS family and TDTS family. (**B**) HMM logos of TDTS and TPTS protein families. (**C**) Fungal STSs from two clades catalyzed cyclization through three different pathways.

To provide an overview of fungal STSs, phylogenetic analysis of the 123 previously reported was performed, resulting in clustering into clades A and B, containing 96 and 27 STSs, respectively (Figure 2A). Further protein family analysis showed that STSs in Clade A had a complete domain of the terpene synthase C (PF19086), while STSs in Clade B did not contain the C domain but had a very special trichodiene synthase domain (PF06330), which has only been reported in fungi (Mistry et al., 2021) (Figure 2B). In addition to protein family differences, the phylogenetic analysis also supported the function divergencies of the STSs from two clades. STSs in Clade A catalyze cyclization through germacrene (C1–C10) and humulene (C1–C11) carbocation intermediates to produce sesquiterpenes such as cadinene, viridiflorene, and protoilludene. However, most STSs from Clade B can catalyze cyclization through a bisabolene (C1–C6) carbocation intermediate to yield sesquiterpenes such as trichodiene, acoradiene, and barbatene (Figure 2C). Due to their sequence and functional differences, we named STSs from Clade A and Clade B as terpene synthase-like sesquiterpene synthases (TPTSs) and trichodiene synthase-like sesquiterpene synthases (TDTSs), respectively.

### 3.2. Extraction and SSN Analysis of TDTSs from a Fungal Genome Database

A comprehensive map was constructed to allow focus on TDTSs with biosynthetic or taxonomic significance. From our in-house fungal genome library, which comprises 430 fungal genomes, 517 TDTS sequences were extracted through two rounds of profile Hidden Markov Model (pHMM) screening. The first round used the TDTS pHMM profile generated from 27 functionally characterized TDTSs to extract potential TDTS sequences (Table S1), while the second round utilized the TPTS pHMM profile generated from 98 functionally characterized TPTSs to exclude the TPTS sequences extracted by the TDTS pHMM (Table S2), and then CD-HIT was used to remove sequences with sequence similarity >80%, resulting in the identification of 517 de-duplicated TDTS sequences. Analysis of genome sequence revealed that TDTS was widely distributed in approximately 54% of species in our in-house 430 members’ fungal genome library. Overall, Basidiomycetes account for the majority, with 58% of TDTSs deriving from Basidiomycetes (305/517) and 42% from Ascomycetes (212/517) (Figures S1 and S2).

**Figure 3:**
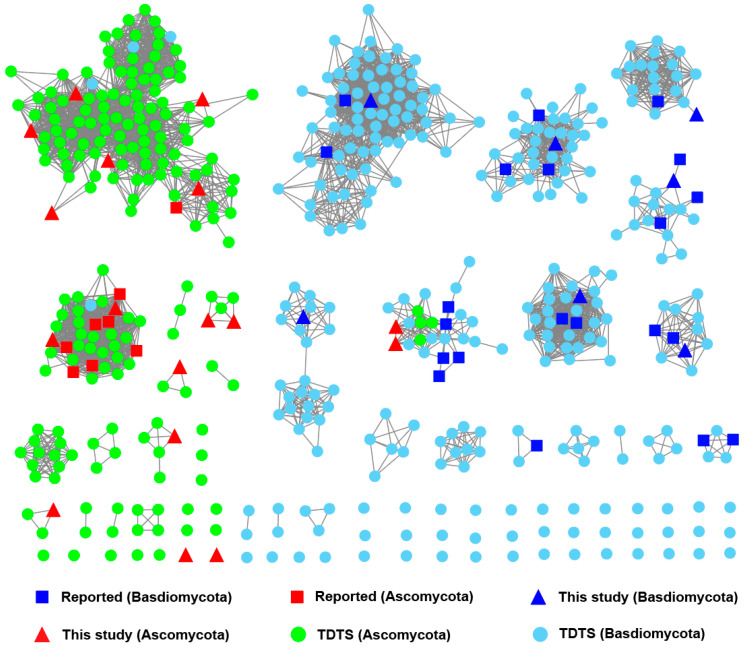
A sequence similarity network of previously reported and extracted fungal TDTSs was constructed with an e-value cut-off set at 10^−75^.

BLAST was used to compute a sequence identity distance matrix for the 517 predicted TDTSs along with the 27 previously reported ones. Utilizing a sequence similarity network with an e-value cut-off set at 10^−75^, the above 545 TDTSs were further defined into 79 families based on sequence identity, giving 2 large families (Families 1 and 3, each with more than 50 TDTSs), 9 medium-sized families (Families 2 and 4–11, each containing 10–50 TDTSs), 10 small families (each with 3–10 TDTSs), and 71 mini families (each with 1–3 TDTSs) (Figure 3). The SSN analysis revealed distinct sequence differences between TDTSs from Ascomycota and Basidiomycota. Most Ascomycota-derived TDTSs (140 out of 212) were classified into Families 1 and 2, whereas most Basidiomycota-derived (228 out of 305) TDTSs were placed in 8 other families (Families 3–10).

### 3.3. Heterologous Expression of TDTSs

These criteria were established for the selection of TDTS for heterologous expression to characterize their function: (1) had a distant evolutionary relationship with the reported TDTS (identity < 60%) (Table S3); (2) contained a complete biosynthetic gene cluster (associated with tailoring enzymes such as P450 monooxygenase and other oxidation-reduction enzymes) (Figures S3 and S4); and (3) possessed complete conservative domains (DDXXD and NSE/DTE motif) (Figure S5). TDTSs were selected for heterologous expression in *A. oryzae* to characterize their function. Twenty-three TDTSs satisfied these criteria.

**Figure 4:**
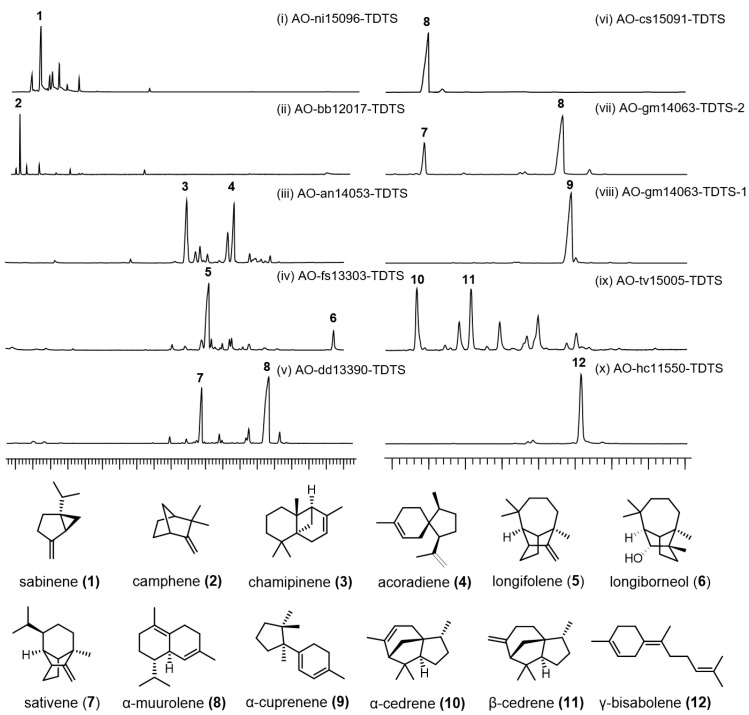
GC–MS profiles of sesquiterpenes produced by *A. oryzae* transformants harbor the selected *TDTSs*. Sesquiterpenes and monoterpenes in the vial headspace were detected by SPME. The structures of compounds detected by GC–MS were identified by comparing their mass spectra with the spectra of terpenoids in the National Institute of Standards and Technology (NIST 14) standard reference database.

SPME-GCMS assays revealed that 10 of the 23 selected TDTSs were activated to produce a series of mono- and sesquiterpenes. Notably, two rare TDTSs, ni15096-TDTS and bb12017-TDTS, produced the monoterpene skeletons sabinene (**1**) and camphene (**2**), respectively (Figure 4i,ii). Although related biosynthetic enzymes have been reported from plants before, this is the first time that such enzymes have been reported from fungi. The remaining eight TDTSs produced sesquiterpene skeletons: chamipinene (**3**), acoradiene (**4**), longifolene (**5**), longiborneol (**6**), sativene (**7**), α-muurolene (**8**), α-cuprenene (**9**), cedrene (**10**, **11**), and γ-bisabolene (**12**) (Figure 4iii–x and Figure S6). Among them, chamipinene (**3**), a rare sesquiterpene, has only been reported from the plant *Illicium oligandrum* [23], with the related biosynthetic enzyme unknown. We report for the first time that an14053-TDTS is the enzyme responsible for catalyzing chamipinene.

### 3.4. Differences in Cyclization Paths of TDTSs in Families 1 and 2

The 27 reported TDTSs, combined with 10 newly functionally characterized ones, were utilized as a guide to investigate the cyclization pathways of each TDTS family. We found that TDTSs from Family 1 and Family 2 catalyze the cyclization of FPP through similar cyclization pathways.

**Figure 5:**
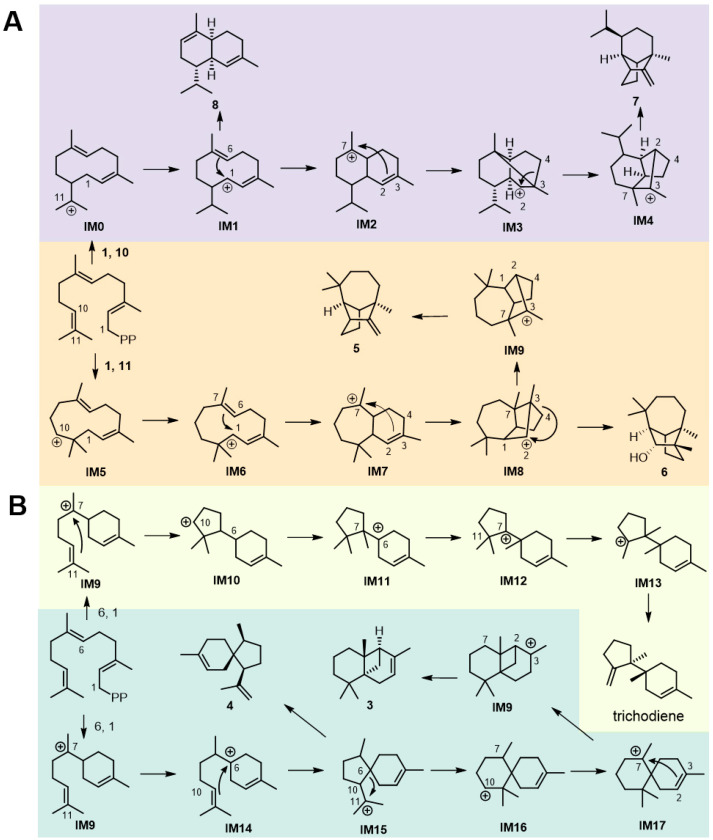
Proposed cyclization mechanism of TDTSs from (**A**) Family 1 and (**B**) Family 2.

Unlike most TDTSs that catalyze cyclization through bisabolene intermediates, TDTSs in Family 1 generate sesquiterpenes **5**, **6**, **7**, and **8** through germacrene and humulene carbocation intermediates. We propose the cyclization pathway for **7** and **8**. Following the diphosphate dissociation from FPP, the newly formed C1–C10 bond results in a tertiary carbocation on C11, creating the germacrene carbocation intermediate **IM0**. Carbocation intermediate **IM0** undergoes a 1,2-hydrogen transfer reaction, with subsequent deprotonation at H7 to yield **8**. While the newly formed C3–C7 bond forms the **IM3** cation, followed by a Wagner–Meerwein rearrangement and deprotonation at C3 to produce **7**. The cyclization of **5** and **6** was similar to that of **7**, with the first cyclization occurring at C1 and C11 instead of at C1 and C10 [24] (Figure 5A).

TDTSs in Family 2 generated sesquiterpenes **3**, **4**, and trichodiene through the bisabolene carbocation intermediate. We propose the cyclization pathway for trichodiene. Following the diphosphate dissociation from FPP, C6 attacks the C1 carbocation to generate the bisabolene carbocation intermediate **IM9**. Intermediate **IM9** undergoes ring closure between C7 and C11, resulting in the **IM10** cation. This intermediate undergoes a 1,4-hydride shift from C10 to C6, and then two successive 1,2-C shifts lead to **IM13**, followed by deprotonation to produce trichodiene. The cyclization pathways of **3** and **4** share the same bisabolene cyclization intermediate, **IM9**, with trichodiene. The newly formed C6–C10 bond yielded the **IM15** cation, followed by deprotonation at C-11 to produce **4**. Intermediate **IM15** underwent a ring expansion and 1,4-hybrid shift to form the **IM16** cation. Subsequent cyclization at C2 and C7 forms the intermediate **IM17**, followed by deprotonation at C3 to yield **3** [25] (Figure 5B).

**Figure 6:**
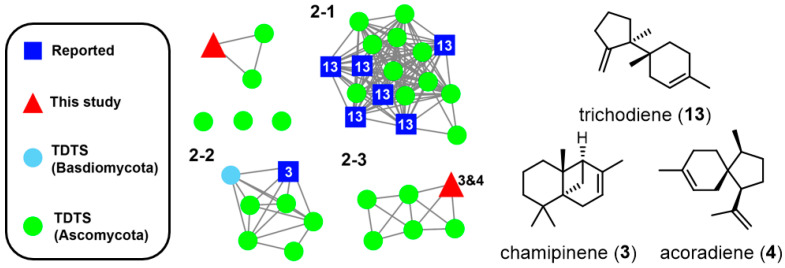
For a more detailed examination of TDTSs within Family 2, a secondary SSN was generated using a different e-value cut-off of 10^−130^.

Within Family 2, certain TDTS synthesized structurally complex sesquiterpenes **3** and **4** via the **IM6** carbocation intermediate, while others produced the common sesquiterpene trichodiene. To distinguish between these two types of TDTS from their sequences, a secondary SSN was generated with a different e-value cut-off of 10^−130^, which indicates that TDTSs in Families 2–1, 2–2, and 2–3 produced trichodiene, **4** and **3** and **4** as major products, respectively (Figure 6).

## 4. Discussion

Fungi offer a rich reservoir for the discovery of novel sesquiterpenoids with diverse bioactivities. Sesquiterpene synthases (STSs) catalyze the cyclization of acyclic FPP precursors to produce a variety of cyclic sesquiterpene scaffolds, which significantly enhance the bioactivity and structural diversity of sesquiterpenoids [3]. The genome mining of fungal STSs could facilitate the discovery of novel sesquiterpenes and enzymes. In this study, phylogenetic analysis of previously reported STSs resulted in the identification of a fungus-specific STS family, trichodiene synthase-like sesquiterpene synthases (TDTSs). The application of HMMs further allowed the discovery of 517 TDTSs from our in-house fungal genome library. Considering the novelty of protein sequences and the completeness of their biosynthetic gene cluster, 23 TDTS genes were selected for biochemical characterization in *Aspergillus oryzae*, leading to the identification of the first chamipinene synthase as well as the first fungal-derived cedrene, sabinene, and camphene synthases. The BGCs of all active TDTSs also contained tailoring enzymes, including cytochromes P450, transferases, and other oxidoreductases (Figure S3). The co-expression of these tailoring enzymes with TDTSs holds promise for generating novel terpenoids.

Monoterpenoids comprise a large terpenoid family widely utilized in the pharmaceutical, food, and perfume industries. While plants are the major known source of monoterpenoids, monoterpenoids from fungi are rare and poorly studied. Only around 70 monoterpenoids have been identified in fungi, accounting for approximately 1% of total fungal terpenoids [1]. In this study, we identified two fungi-derived MTSs, sabinene and camphene synthases. Although several sabinene synthases [26] and camphene synthases [27] have been identified in plants, none have been characterized in fungi prior to this study. Sabinene finds applications as flavorings, perfume additives, fine chemicals, and advanced biofuels. Similarly, camphene can be used as a food additive and fragrance component [26]. Our work not only broadens the diversity of known fungus-derived MTSs but also presents new avenues for the biological production of these valuable compounds. The further co-expression of TDTSs and modified genes in biosynthetic gene clusters should also provide insights into the biosynthesis of this valuable terpenoid family. Among the five functionally characterized MTSs, four belong to the TDTS family, suggesting the substantial potential of TDTSs to produce monoterpenes.

*Trichoderma* species are renowned for their ability to colonize the root surface and promote plant growth [28]. Previous research has indicated that cedrene, produced by *Trichoderma guizhouense*, can modulate *Arabidopsis* root development through the transport and signaling of auxin [29]. However, the fungal STS responsible for cedrene production remains unknown. In this study, the first fungal cedrene synthases were characterized from *Trichoderma voglmayr*, offering potential opportunities for developing *Trichoderma* strains with agricultural applications.

Recent advancements in genome mining of fungal STSs have unveiled numerous novel sesquiterpenes and enzymes [7]. However, the sequence-function relationships of STSs have been barely studied. In this study, guided by 38 functionally characterized TDTSs, we explored the sequence-function relationships among different TDTS families. TDTSs in Family 1 could produce bridged cyclic sesquiterpenes such as **5**, **6**, and **7**, while those in Family 2 could synthesize spiro and bridged cyclic sesquiterpenes such as **3** and **4**. Further genome mining of these two TDTS families holds the potential to unveil additional complex polycyclic sesquiterpenes.

In summary, in this study, through the phylogenetic analysis of 123 reported fungal STSs, we identified a fungi-specific STS family, TDTS. The systematic screening of TDTSs from our in-house genome library and the further SSN analysis provided a comprehensive landscape of TDTSs. Considering the novelty of protein sequences and the completeness of their BGCs, 23 TDTS genes were selected for biochemical characterization in *A. oryzae*, resulting in the identification of the first chamipinene synthase as well as the first fungal cedrene, sabinene, and camphene synthases. Additionally, with the guidance of 38 functionally characterized TDTSs, we made preliminary speculation regarding the potential sequence-function relationships of TDTSs from Ascomycota. Our research presents a new avenue for the genome mining of fungal sesquiterpenoids.

## Data Availability

All 10 active STS protein sequences are available in the National Center for Biotechnology Information under the accession number PP516626-35. The 13 inactive *TDTS* sequences deposited in the National Center for Biotechnology Information under accession number PP776614-26.

## Supplementary Materials

The following supporting information can be downloaded at https://www.mdpi.com/article/10.3390/jof10050350/s1. Figure S1: Number of TDTSs by genome size; Figure S2: Density of TDTSs in different fungi classES; Figure S3: BGCs of active TDTSs; Figure S4: BGCs of inactive; Figure S5: Alignment of reported TDTSs and selected TDTSs in this study; Figure S6: The mass spectra of all sesquiterpene skeletons in this study. Table S1: Reported fungal TDTSs collected from a public database and published papers; Table S2: Reported fungal TPTSs collected from a public database and published papers; Table S3: Primers used in this study; Table S4: Plasmids and transformants constructed in this study. Table S5: TDTSs selected for heterologous expression. References [8,9,10,11,30,31,32,33,34,35,36,37,38,39,40,41,42,43,44,45,46,47,48,49,50,51,52,53,54,55,56,57,58,59,60] are cited in the Supplementary Materials.
