# Supplementary material for: Genome Mining of Fungal Unique Trichodiene Synthase-like Sesquiterpene Synthases
Source: J Fungi (Basel). 2024 May 13;10(5):350. doi: 10.3390/jof10050350 (PMC11122449; doi:10.3390/jof10050350)
Supplement: Supplementary file 1 [file jof-10-00350-s001.zip › jof-2990946-supplementary.pdf]

Journal of Fungi

Supplementary material

Genome mining of fungal unique trichodiene synthase-like sesquiterpene synthases

Zhanren Cong[1], Qiang Yin[1], Kunhong Tian[1], Njeru Joe Mukoma[1], Liming Ouyang[1], Tom Hsiang[2], Lixin Zhang[1], Lan Jiang[3,*] and Xueting Liu[1,*]

[1]State Key Laboratory of Bioreactor Engineering, East China University of Science of Technology, Shanghai 200237, China

[2]School of Environmental Sciences, University of Guelph, 50 Stone Road East, Guelph, Ontario N1G2W1, Canada

[3]Department of Cardiothoracic Surgery, Children's Hospital of Nanjing Medical University, Nanjing 210093, China

*Corresponding authors:

Xueting Liu, E-mail: liuxueting@ecust.edu.cn;

Lan Jiang, E-mail: jianglan0426@163.com

Contents

Supplementary Text 3
Sequence of pUARA4.. 3
Supplementary Figures 7
Figure S1. Number of TDTSs by genome size.. 7
Figure S2. Density of TDTSs in different fungi class.. 7
Figure S3. BGCs of active TDTSs. 8
Figure S4. BGCs of inactive TDTSs.. 8
Figure S5. Alignment of reported TDTSs and selected TDTSs in this study. 9
Figure S6. The mass spectra of all sesquiterpene skeletons in this study. 10
Supplementary Tables 11
Table S1. Reported fungal TDTSs extracted from public database and PubMed. 11
Table S2. Reported fungal TPTSs extracted from public database and PubMed. 12
Table S3. TDTSs selected for heterologous expression. 15
Table S4. Primers used in this study. 16
Table S5. Plasmids and transformants constructed in this study. 18

Supplementary Text

Sequence of pUARA4

AGCGCCCAATACGCAAACCGCCTCTCCCCGCGCGTTGGCCGATTCATTAAT
GCAGCTGGCACGACAGGTTTCCCGACTGGAAAGCGGGCAGTGAGCGCAA
CGCAATTAATGTGAGTTAGCTCACTCATTAGGCACCCCAGGCTTTACACTTT
ATGCTTCCGGCTCGTATGTTGTGTGGAATTGTGAGCGGATAACAATTTCACA
CAGGAAACAGCTATGACCATGATTACGATATCATGGTGTTTTGATCATTTTA
AATTTTTATATGGCGGGTGGTGGGCAACTCGCTTGCGCGGGCAACTCGCTT
ACCGATTACGTTAGGGCTGATATTTACGTAAAAATCGTCAAGGGATGCAAG
ACCAAAGTAGTAAAACCCCGGAGTCAACAGCATCCAAGCCCAAGTCCTTC
ACGGAGAAACCCCAGCGTCCACATCACGAGCgaaggaccacctctaggcatcggacgcac
catCCAATTAGAAGCAGCAAAGCGAAACAGCCCAAGAAAAAGGTCGGCCCG
TCGGCCTTTTCTGCAACGCTGATCACGGGCAGCGATCCAACCAACACCCTC
CAGAGTGACTAGGGGCGGAAATTTAAAGGGATTAATTTCCACTCAACCACA
AATCACAGTCGTCCCCGGTATTGTCCTGCAGAATGCAATTTAAACTCTTCTG
CGAATCGCTTGGATTCCCCGCCCCTGGCCGTAGAGCTTAAAGTATGTCCCTT
GTCGATGCGATGTATCACAACATATAAATACTAGCAAGGGATGCCATGCTTG
GAGGATAGCAACCGACAACATCACATCAAGCTCTCCCTTCTCTGAACAATA
AACCCCACAGcaagctccgAATTCGAGCTCGGTACCCGGGGATCtgtagtagctcgtgaa
GGGTGGAGAGTATATGATGGTACTGCTATTCAATCTGGCATTGGACAGTGAG
TTTGAGTTTGATGTACAGTTGGAGTCGTTACTGCTGTCATCCCCTTATACTCT
TCGATTGTTTTTCGAACCCTAACGCCAAGCACGCTAGTCTATTATAGGAAAG
GATcCAGGAAACAGCTATGACCATGATTACGATATCATGGTGTTTTGATCATT
TTAAATTTTTATATGGCGGGTGGTGGGCAACTCGCTTGCGCGGGCAACTCG
CTTACCGATTACGTTAGGGCTGATATTTACGTAAAAATCGTCAAGGGATGCA
AGACCAAAGTAGTAAAACCCCGGAGTCAACAGCATCCAAGCCCAAGTCCT
TCACGGAGAAACCCCAGCGTCCACATCACGAGCgaaggaccacctctaggcatcggacgc
accatccaattagaagcagcaaagcgaaacagcccaagaaaaaggtcggcccgtcggccttttctgcaacgctgatcacg
ggcagcgatccaaccaacaccctccagagtgactaggggcggaaatttaaagggattaatttccactcaaccacaaatcac
agtcgtccccggtattgtcctgcagaatgcaatttaaactcttctgcgaatcgcttggattccccgcccctggccgtagagctt
aaagtatgtcccttgtcgatgcgatgtatcacaacatataaatactagcaagggatgccatgcttggaggatagcaaccgac
aacatcacatcaagctctcccttctctgaacaataaaccccacagcaagctccgTTAATTAAATTTAAATcatat
gcacgtggggtggagagtatatgatggtactgctattcaatctggcattggacagtgagtttgagtttgatgtacagttggagt
cgttactgctgtcatccccttatactcttcgattgtttttcgaaccctAACGCCAAGCACGCTAGTCTATTA
TAGGAAAGGATcccaggaaacagctatgaccatgattacgatatcatggtgttttgatcattttaaatttttatatggc
gggtggtgggcaactcgcttgcgcgggcaactcgcttaccgattacgttagggctgatatttacgtaaaaatcgtcaaggga
tgcaagaccaaagtagtaaaaccccggagtcaacagcatccaagcccaagtccttcacggagaaaccccagcgtccaca
tcacgagcgaaggaccacctctaggcatcggacgcaccatCCAATTAGAAGCAGCAAAGCGAAA
CAGCCCAAGAAAAAGGTCGGCCCGTCGGCCTTTTCTGCAACGCTGATCAC
GGGCAGCGATCCAACCAACACCCTCCAGAGTGACTAGGGGCGGAAATTTA
AAGGGATTAATTTCCACTCAACCACAAATCACAGTCGTCCCCGGTATTGTC
CTGCAGAATGCAATTTAAACTCTTCTGCGAATCGCTTGGATTCCCCGCCCCT

GGCCGTAGAGCTTAAAGTATGTCCCTTGTCGATGCGATGTATCACAACATAT
AAATACTAGCAAGGGATGCCATGCTTGGAGGATAGCAACCGACAACATCAC
ATCAAGCTCTCCCTTCTCTGAACAATAAACCCCACAGCAAGCTCCGAATTC
GAATCGATTTGAGCTAGCGGCCTCGTACCCGATGATGAAACATAATGGACA
AGTTGGTGAAAGGTTGGGCCAGAGGTTGGACGGTCGTGAGGGTTATATTG
GGGACGGTGAATATATATAGGAACATCGTTCCTTATAGTGCGGATGGTGTGG
TAAGTATAGACTTTGGATGAAATTCGTTTTTGGATAATTGTGTCAGTATATGA
TTTGGTGTGGTCTTTTCGTTTGATTTTAGCATAGGTGTTATGGGACGTTAAG
ACTCTTCCACCCAGGAAACAGCTATGACCATGATTACGATATCATGGTGTTT
TGATCATTTTAAATTTTTATATGGCGGGTGGTGGGCAACTCGCTTGCGCGGG
CAACTCGCTTACCGATTACGTTAGGGCTGATATTTACGTAAAAATCGTCAAG
GGATGCAAGACCAAAGTAGTAAAACCCCGGAGTCAACAGCATCCAAGCCC
AAGTCCTTCACGGAGAAACCCCAGCGTCCACATCACGAGCgaaggaccacctctag
gcatcggacgcaccatccaattagaagcagcaaagcgaaacagcccaagaaaaaggtcggcccgtcggccttttctgca
acgctgatcacgggcagcgatccaaccaacaccctccagagtgactaggggcggaaatttaaagggattaatttccactca
accacaaatcacagtcgtccccggtattgtcctgcagaatgcaatttaaactcttctgcgaatcgcttggattccccgcccctg
gccgtagagcttaaagtatgtcccttgtcgatgcgatgtatcacaacatataaatactagcaagggatgccatgcttggagga
tagcaaccgacaacatcacatcaagctctcccttctctgaacaataaaccccacagcaagctccgGGCCGCACTA
GTGACCCGGGTAGTCGTACCCGATGATGAAACATAATGGACAAGTTGGTGA
AAGGTTGGGCCAGAGGTTGGACGGTCGTGAGGGTTATATTGGGGACGGTG
AATATATATAGGAACATCGTTCCTTATAGTGCGGATGGTGTGGTAAGTATAGA
CTTTGGATGAAATTCGTTTTTGGATAATTGTGTCAGTATATGATTTGGTGTGG
TCTTTTCGTTTGATTTTAGCATAGGTGTTATGGGACGTTAAGACTCTTCCAC
CAAGAATAAATGTATTCTTGTATACTACAAAAGTACCAAAATACTCCAATGA
GGAGTCTCGGTTAGGATAACTAACCAGAACGGCAGATCCTCTAGACTGATG
ATGCAGTGGTTCGTTCACATTCTGCTCTTGAAGATTGGAGTCGCTAAAATG
AATAGGAGTTGAGCTCTTGCTTCGGGTGTGGTGGCGGGCGCGTGAGCCAC
TCCGGGAAGGCGCACGAGAGAGGACGCCGGGTGTCCCTGGGACCGAATA
GCTAGACAAGTAGGAAGTTCTAGTGGGGTTGTCAATAGCCTCATTTCGCGG
GGTGAAATAATCCTGTGGGTCTTTGTCCGACTCATTGTCGTCGTCGTCAATG
GGGTAATGGGGAGTTAAAGGCGCGAGTGAGATGCGGTTTAAGGATGGATA
AGAGCGGCGGGGCCGAGCTTGTGATGCCGAGGACTGTTCAGCTTTGTTAG
CGAATCAGCAGGATTGTGGATGTAGTAAAgtcgacctacagccattgcgaaacctcagaagaat
gtgctggatagaggtcaagagtatggaatgaggagagtcgagggttggggtgtgttaggcctggatctaccttggctagga
cgactccgcattcgtgacatgtgaaagtcaccgggtgcgatttgccccatttccaatactctgagtaatataaggaagcgag
agaacatgtcaattagttatttgatatatgctagaaagaattggtcaggataattgggaaaatgcttctactaacaggcttttacg
tgatcggagtaaatatatttagggacggaatgatctcatacccgtcaactcatcagtttgataacgccagcctctatatatttaa
atggaagcctggttatatttattcaatttttcccttattgacaacgaaaccctccaaggcggaaatcgcagcccataaccggttc
tcagcctcagggaagacaagtgaccggttgctgtagaaaacctcgtcgctgacctcctcagggtgtcgcgggagacagtg
catgaacttccagccctccttagctcctcctcgcttggcgagttcagcagtgatttggaaaccatcaaactccttcagcctctg
agccttctcttcctcttggcccatagagacccaggtgtctgtgaccagaatatcggcgtccttgaccgcttcttcgggaatatt
ggtttgcagaagctttcctggtttcgagacaccctctccagcagacttgatgagctccagcatgtgaggagggatttcgtacc
ccttgggagtagcgacagcaatgtcgacacccatttttgtagcagcaatggccatatcgaacaggacgttgttggcgtcacc
cacccaagcgatcttcaatccttccaaccctagacttgaaaggtggtgcgccttgggggtgaatgcttcatagatggtctgga

aatcggccacggcttggagagggtggaaagagtcacacaaagcattgatgactggaaccgaagagtgcttcgccagatct
gcgacctctgcatgtttaccgacacgggctacaatgcaggataccatggacgaaatgacaacggaggtgtcgtataggga
ctcgttgacacctagttggatatcatccttgcccaagaacatcggatgacctcccatctgcacaacggccccttctgtagata
cccttgtcctcgtgcttcgtttgctgaagatcatggccacagttttcccaaggagtgatccctgcaagttttggggcattgaccc
cgacttgatagcccgtttgtgtgaggaggcattgcgaacaagggttgcgaactcggagggcgagaggtccgcaatggag
aggaagtggcggggagcaaagggtgaggcgggaggcatggtttgcgaggagtaggatcgcacggtgtgtcgcaagct
ctggctcttgagtacggagcgaagggatgccattggaagtgcaagaatatagatgttaaagaacttaaaagacttggggcg
gaaagaaattgtggacagaacgagggcatccacaaataactgagaaatgattcgtgaatgactcataagtccgccagcag
gagcttccgatcattggtaaagaaacagagttagcgccccttccgttcgctgatcacggatcaccgccattgcatcaggccc
ttgactctgctgataccttgaactgctcacagtccttcatcgtcctacctctcgcttcatcaatatttgctgcaattattgcatgcgt
tcatctctatttatctaatttatgtaaattatagtcaaatgaggcctctaaactggtcatatctggcccgattcgccgtctgatcag
cctcaacactatcgcatactctccacaatccacactttccccccactccgctttacacagcgttcgttatgacggatgaggc
gagtcggcctctgttgggcgagcatcacgacaataatgcttctccctatcaagcgtcgtctacaaatcaacagcgtcgctcct
ttgaagtgtcttcggagtcaaccccacttctccatcgccgcgatgatgatatatcggcgtacggtggtatagaGTCATC
GCGGGCTTCCTCTGCTGCGTCCGGGTCTGTTTCATTAGATGACTACCCCAA
AAAACCGCGAAATAAAGCTTGGTGGACGGTATTATGCGGCCTCCTTGCTGT
CGGTTCCGTCATTGTCATCCTCATTCTGGCCTTCTTTGTTCCCGAGGTAGTA
AAGCAATACGTCAAGGAAGCAGCCATATTTAGACCGACAAACCTATCAGTC
GTGTCGGCCACAAGCGAAGGTGTCAGGGCAAGGGTACAAGGGGATATTGT
CCTCGATGCTGATCGAGTCAAAAGTGAATCGGCGAGAAATATTGGGCGTTT
CGTCACCTGGATTGGGAAAGAGGTCGAAACCGGCCAGTCTGAAGTAATTG
TACGCTTACCGGAATACGAAAATGCCCTTGTTGGATCTGCATCCTTGCCTTC
CATCAAGGTTAGCATTCGCAACGGCCATGTGAATCACCTTGACTTCGAAGC
CGATTTGATTGCTGGCGACATTGAGGGCTTGCGCTCCGTCGCCGTCGACTG
GCTGGAAGGGAGACTGGACCGTCTATTACTTCATGGAAGTGTCATTCTCCAT
ATCAAATCAGGCCTGCTGAGCCTGGGAGAGCAGACTCTAGAGTCGACCTG
CAGGCATGCAAGCTTGGCACTGGCCGTCGTTTTACAACGTCGTGACTGGGA
AAACCCTGGCGTTACCCAACTTAATCGCCTTGCAGCACATCCCCCTTTCGC
CAGCTGGCGTAATAGCGAAGAGGCCCGCACCGATCGCCCTTCCCAACAGTT
GCGCAGCCTGAATGGCGAATGGCGCCTGATGCGGTATTTTCTCCTTACGCAT
CTGTGCGGTATTTCACACCGCATACGTCAAAGCAACCATAGTACGCGCCCT
GTAGCGGCGCATTAAGCGCGGCGGGTGTGGTGGTTACGCGCAGCGTGACC
GCTACACTTGCCAGCGCCCTAGCGCCCGCTCCTTTCGCTTTCTTCCCTTCCT
TTCTCGCCACGTTCGCCGGCTTTCCCCGTCAAGCTCTAAATCGGGGGCTCC
CTTTAGGGTTCCGATTTAGTGCTTTACGGCACCTCGACCCCAAAAAACTTG
ATTTGGGTGATGGTTCACGTAGTGGGCCATCGCCCTGATAGACGGTTTTTCG
CCCTTTGACGTTGGAGTCCACGTTCTTTAATAGTGGACTCTTGTTCCAAACT
GGAACAACACTCAACCCTATCTCGGGCTATTCTTTTGATTTATAAGGGATTT
TGCCGATTTCGGCCTATTGGTTAAAAAATGAGCTGATTTAACAAAAATTTAA
CGCGAATTTTAACAAAATATTAACGTTTACAATTTTATGGTGCACTCTCAGT
ACAATCTGCTCTGATGCCGCATAGTTAAGCCAGCCCCGACACCCGCCAACA
CCCGCTGACGCGCCCTGACGGGCTTGTCTGCTCCCGGCATCCGCTTACAGA
CAAGCTGTGACCGTCTCCGGGAGCTGCATGTGTCAGAGGTTTTCACCGTCA
TCACCGAAACGCGCGAGACGAAAGGGCCTCGTGATACGCCTATTTTTATAG

GTTAATGTCATGATAATAATGGTTTCTTAGACGTCAGGTGGCACTTTTCGGG
GAAATGTGCGCGGAACCCCTATTTGTTTATTTTTCTAAATACATTCAAATATG
TATCCGCTCATGAGACAATAACCCTGATAAATGCTTCAATAATATTGAAAAA
GGAAGAGTATGAGTATTCAACATTTCCGTGTCGCCCTTATTCCCTTTTTTGC
GGCATTTTGCCTTCCTGTTTTTGCTCACCCAGAAACGCTGGTGAAAGTAAA
AGATGCTGAAGATCAGTTGGGTGCACGAGTGGGTTACATCGAACTGGATCT
CAACAGCGGTAAGATCCTTGAGAGTTTTCGCCCCGAAGAACGTTTTCCAAT
GATGAGCACTTTTAAAGTTCTGCTATGTGGCGCGGTATTATCCCGTATTGAC
GCCGGGCAAGAGCAACTCGGTCGCCGCATACACTATTCTCAGAATGACTTG
GTTGAGTACTCACCAGTCACAGAAAAGCATCTTACGGATGGCATGACAGTA
AGAGAATTATGCAGTGCTGCCATAACCATGAGTGATAACACTGCGGCCAAC
TTACTTCTGACAACGATCGGAGGACCGAAGGAGCTAACCGCTTTTTTGCAC
AACATGGGGGATCATGTAACTCGCCTTGATCGTTGGGAACCGGAGCTGAAT
GAAGCCATACCAAACGACGAGCGTGACACCACGATGCCTGTAGCAATGGC
AACAACGTTGCGCAAACTATTAACTGGCGAACTACTTACTCTAGCTTCCCG
GCAACAATTAATAGACTGGATGGAGGCGGATAAAGTTGCAGGACCACTTCT
GCGCTCGGCCCTTCCGGCTGGCTGGTTTATTGCTGATAAATCTGGAGCCGG
TGAGCGTGGGTCTCGCGGTATCATTGCAGCACTGGGGCCAGATGGTAAGCC
CTCCCGTATCGTAGTTATCTACACGACGGGGAGTCAGGCAACTATGGATGAACGA
AATAGACAGATCGCTGAGATAGGTGCCTCACTGATTAAGCATTGGTAACTGTCAGACCA
AGTTTACTCATATATACTTTAGATTGATTTAAAACTTCATTTTTAATTTAAAAGGATCTAG
GTGAAGATCCTTTTTGATAATCTCATGACCAAAATCCCTTAACGTGAGTTTTCGTTCCAC
TGAGCGTCAGACCCCGTAGAAAAGATCAAAGGATCTTCTTGAGATCCTTTTTTTCTGCG
CGTAATCTGCTGCTTGCAAACAAAAAAACCACCGCTACCAGCGGTGGTTTGTTTGCCG
GATCAAGAGCTACCAACTCTTTTTCCGAAGGTAACTGGCTTCAGCAGAGCGCAGATAC
CAAATACTGTCCTTCTAGTGTAGCCGTAGTTAGGCCACCACTTCAAGAACTCTGTAGCA
CCGCCTACATACCTCGCTCTGCTAATCCTGTTACCAGTGGCTGCTGCCAGTGGCGATAA
GTCGTGTCTTACCGGGTTGGACTCAAGACGATAGTTACCGGATAAGGCGCAGCGGTCG
GGCTGAACGGGGGGTTCGTGCACACAGCCCAGCTTGGAGCGAACGACCTACACCGAA
CTGAGATACCTACAGCGTGAGCTATGAGAAAGCGCCACGCTTCCCGAAGGGAGAAAG
GCGGACAGGTATCCGGTAAGCGGCAGGGTCGGAACAGGAGAGCGCACGAGGGAGCT
TCCAGGGGGAAACGCCTGGTATCTTTATAGTCCTGTCGGGTTTCGCCACCTCTGACTTG
AGCGTCGATTTTTGTGATGCTCGTCAGGGGGGCGGAGCCTATGGAAAAACGCCAGCAA
CGCGGCCTTTTTACGGTTCCTGGCCTTTTGCTGGCCTTTTGCTCACATGTTCTTTCCTGC
GTTATCCCCTGATTCTGTGGATAACCGTATTACCGCCTTTGAGTGAGCTGATACCGCTCG
CCGCAGCCGAACGACCGAGCGCAGCGAGTCAGTGAGCGAGGAAGCGGAAG

Supplementary Figures

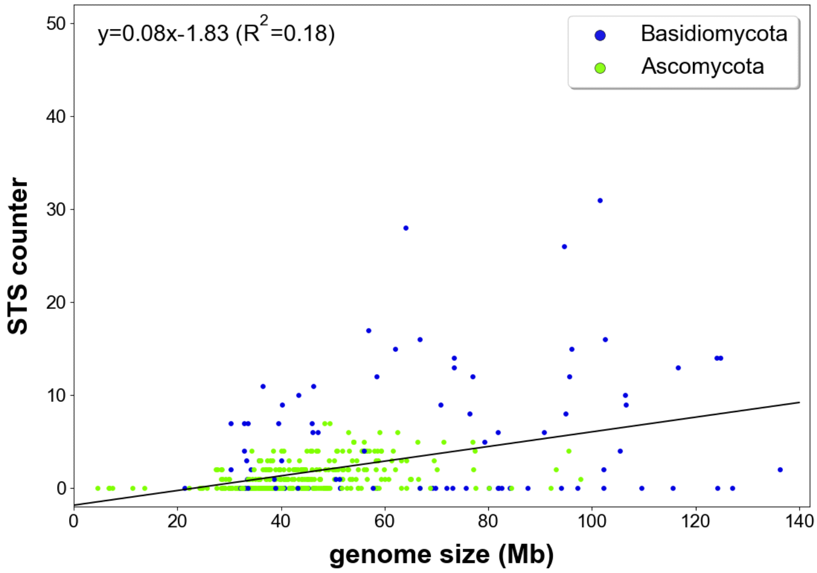

Figure S1. Number of TDTSs by genome size. Each point represented one fungi strain. Strains from Basidiomycota were labeled as blue. Strains from Ascomycota were labeled as green.

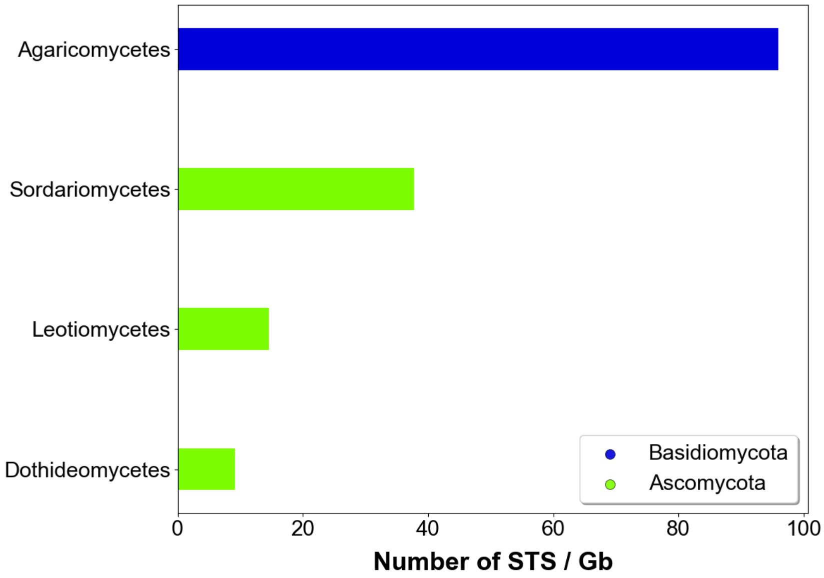

Figure S2. Density of TDTSs in different fungi class. Class from Basidiomycota were labeled as blue. Class from Ascomycota were labeled as green.

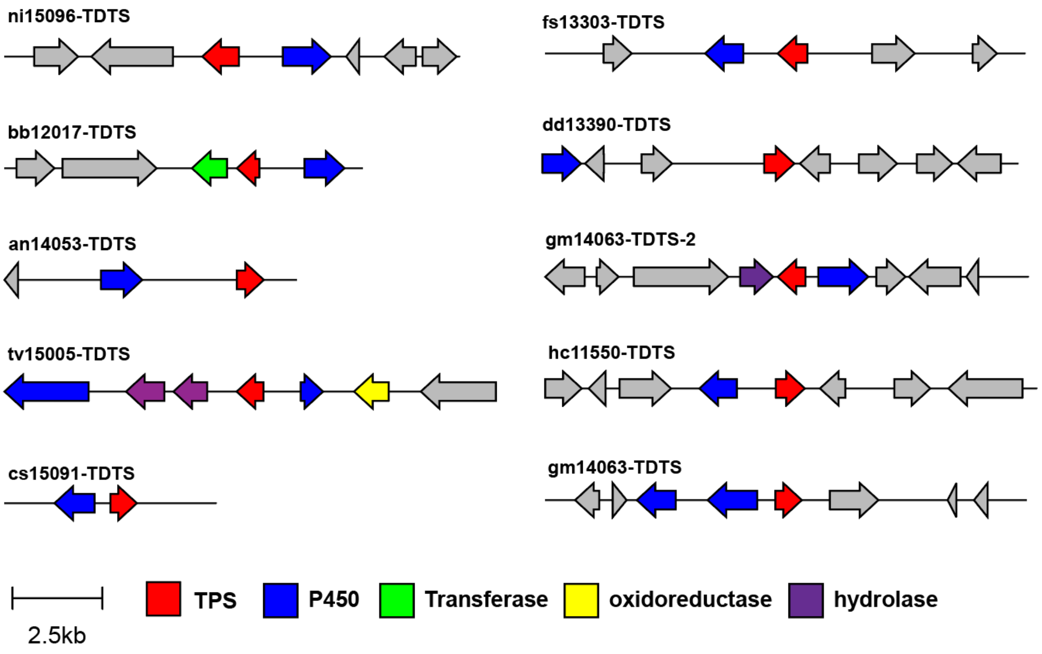

Figure S3. BGCs of active TDTSs.

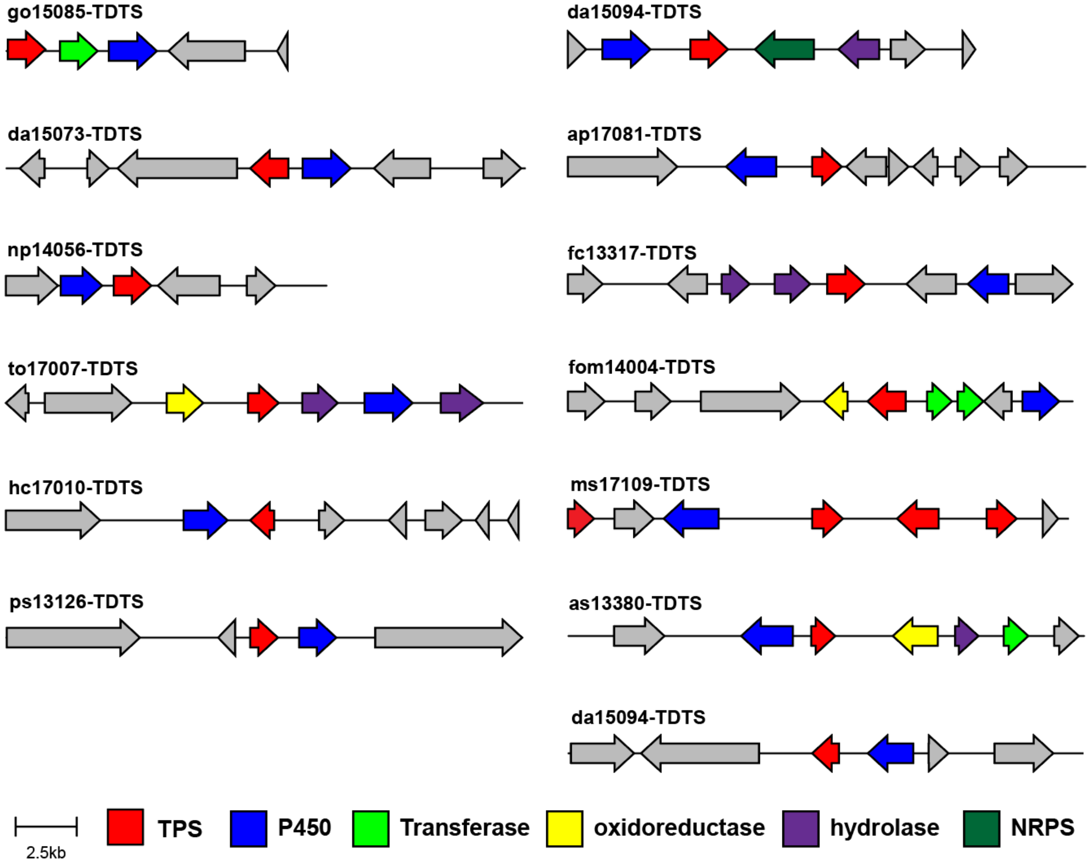

Figure S4. BGCs of inactive TDTSs.

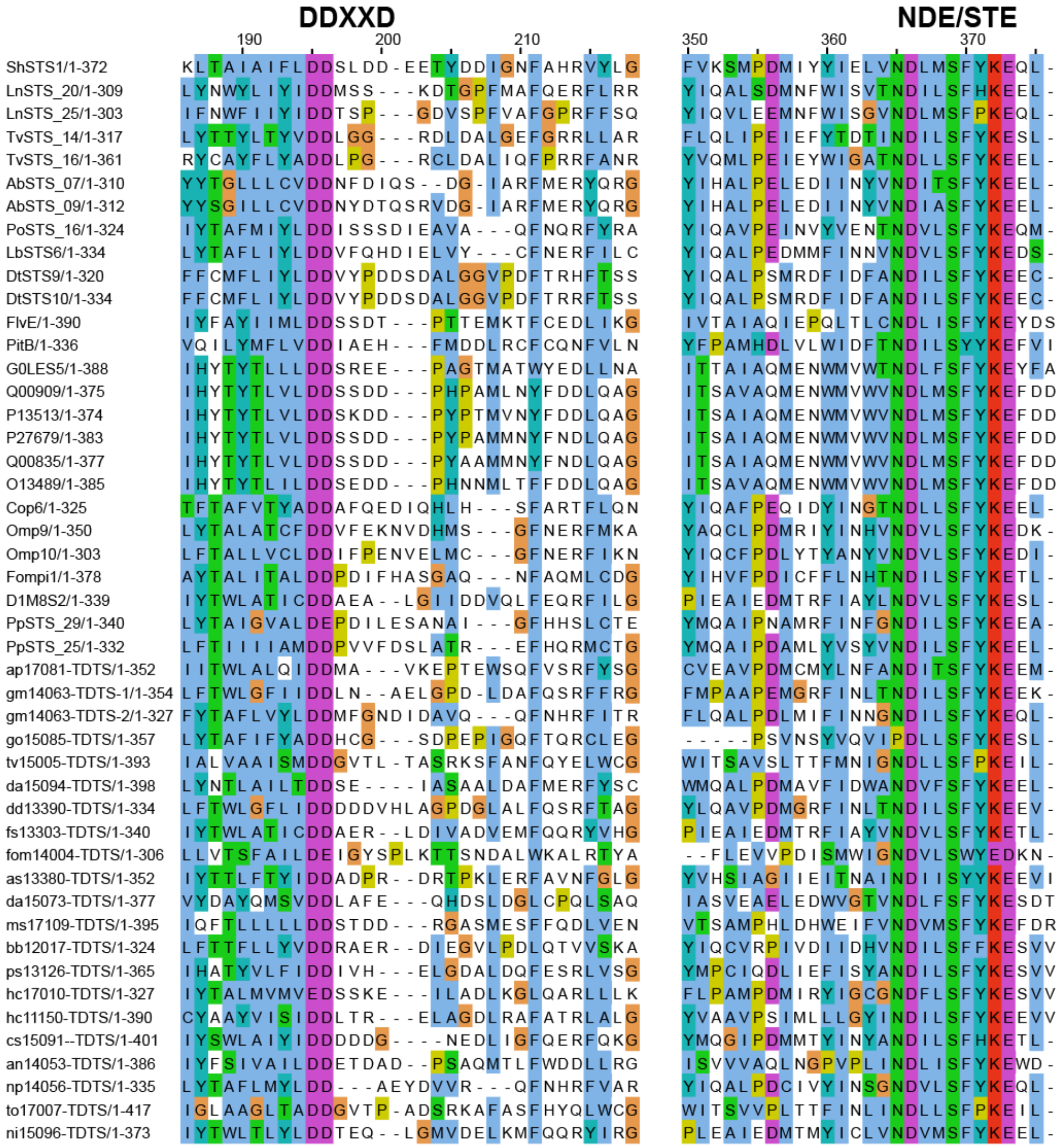

Figure S5. Alignment of reported TDTSs and selected TDTSs in this study.

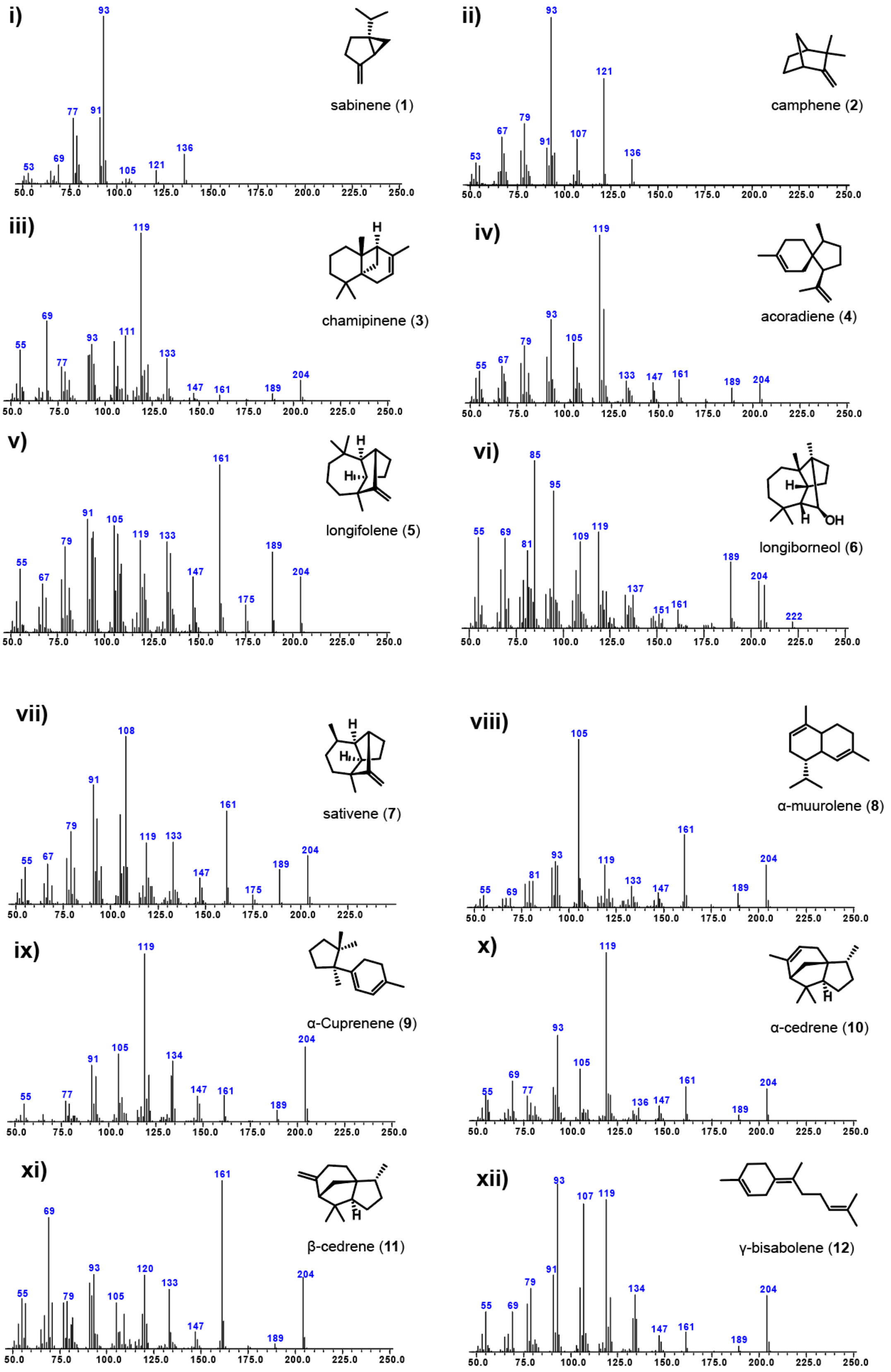

Figure S6. The mass spectra of all sesquiterpene skeletons in this study.

Supplementary Tables

Table S1. Reported fungal TDTSs extracted from public database and PubMed.

Accession	ID	Organism	Product	Intermediate	Ref.
PubMed	DtSTS09	*Desarmillaria tabescens*	Cis-thujosene	Bisabolene	[1]
PubMed	DtSTS10	*Desarmillaria tabescens*	α-Cuprenene	Bisabolene	
KAG2008248	Cop6	*Coprinus cinereus*	α-cuprenene	Bisabolene	[2]
JGI ID: 3258	Omp9	*Omphalotus olearius*	α-Barbatene	Bisabolene	[3]
JGI ID: 3981	Omp10	*Omphalotus olearius*	Dauca-4(11),8-diene	others	
PubMed	ShSTS1	*Stereum hirsutum*	β-barbatene	Bisabolene	[4]
JGI ID: 84944	Fompi1	*Fomitopsis schrenkii*	α-Cuprenene	Bisabolene	[3]
PubMed	TvSTS14	*Trametes versicolor*	β-barbatene	Bisabolene	[5]
PubMed	TvSTS16	*Trametes versicolor*	Dauca-4(11),8-diene	others	
PubMed	LnSTS20	*Lepista nuda*	β-barbatene	Bisabolene	
PubMed	AbSTS09	*Agaricus bisporus*	(Z)-α-bisabolene	Bisabolene	
PubMed	AbSTS07	*Agaricus bisporus*	δ-Cadinene	germacrene	
PubMed	PoSTS16	*Pleurotus ostreatus*	α-Cuprenene	Bisabolene	
I1S104.1	D1M8S2	*Fusarium graminearum*	Longiborneol	Humulene	[6]
B8NHE0.1	FlvE	*Aspergillus flavus*	Acoradiene	Bisabolene	[7]
XP_001885710	LbSTS6	*Laccaria bicolor*	α-Cuprenene	Bisabolene	[8]
Q00909.1	Q00909	*Fusarium graminearum*	Trichodiene	Bisabolene	[9]
G0LES5.1	G0LES5	*Trichoderma arundinaceum*	Trichodiene	Bisabolene	[9]
P13513.1	P13513	*Trichoderma arundinaceum*	Trichodiene	Bisabolene	[9]
P27679.1	P27679	*Gibberella pulicaris*	Trichodiene	Bisabolene	[9]
Q00835.1	Q00835	*Fusarium poae*	Trichodiene	Bisabolene	[9]
O13489.1	O13489	*Paramyrothecium roridum*	Trichodiene	Bisabolene	[9]
A0A348B793.1	PpSTS25	*Postia placenta*	linalool	others	[10]
KGO75161.1	PitB	*Penicillium italicum*	Nerol	others	[11]
PubMed	PcSTS06	*Phanerodontia chrysosporium*	a-barbatene	Bisabolen	[12]
PubMed	PcSTS08	*Phanerodontia chrysosporium*	α-bisabolene	Bisabolen	[12]
PubMed	PcSTS11	*Phanerodontia chrysosporium*	santalene	Bisabolen	[12]

Table S2. Reported fungal TPTSs extracted from public database and PubMed.

Accession	ID	Organism	Major product	Intermediate	Ref.
**Basdiomycota**					
QDF59313.1	Hfas94a	*Hypholoma fasciculare*	humulene	humulene	[13]
QDF59314.1	Hfas94b	*Hypholoma fasciculare*	caryophyllene	humulene	
QDF59315.1	Hfas344	*Hypholoma fasciculare*	humulene	humulene	
PubMed	STC9	*Termitomyces sp. J132*	(–)-gamma-cadinene	germacrene	[14]
PubMed	STC15	*Termitomyces sp. J132*	Germacrene-D-4-ol	germacrene	
PubMed	STC4	*Termitomyces sp. J132*	Intermedeol	germacrene	
BBD74522.1	PpSTS08	*Postia placenta*	delta-6-protoilludene	humulene	[15]
JGI ID: 99496	PpSTS03	*Postia placenta*	alpha-Cadinene	germacrene	
BBD74520.1	PpSTS06	*Postia placenta*	alpha-Gurjunene	germacrene	
JGI ID: 98072	PpSTS10	*Postia placenta*	delta-Cadinene	germacrene	
BBD74517.1	PpSTS01	*Postia placenta*	alpha-Muurolene	germacrene	
JGI ID: 101549	PpSTS14	*Postia placenta*	pentalenene	humulene	
XP_007765978	Copu3	*Coniophora puteana*	Cubebol	germacrene	[16]
XP_007771895	Copu2	*Coniophora puteana*	beta-Copaene	germacrene	
KX281943.1	GME6364	*Lignosus rhinocerotis*	(+)-torreyol	germacrene	[17]
KX281944.1	GME6368	*Lignosus rhinocerotis*	alpha-cadinol	germacrene	
JGI ID: 1311	Omp1	*Omphalotus olearius*	alpha-Muurolene	germacrene	[3]
JGI ID: 1447	Omp4	*Omphalotus olearius*	delta-cadinene	germacrene	
JGI ID: 2271	Omp7	*Omphalotus olearius*	delta-6-protoilludene	humulene	
JGI ID: 2392	Omp5a	*Omphalotus olearius*	gamma-cadinene	germacrene	
JGI ID: 2393	Omp5b	*Omphalotus olearius*	gamma-cadinene	germacrene	
JGI ID: 4636	Omp3	*Omphalotus olearius*	alpha-Muurolene	germacrene	
JGI ID: 4774	Omp6	*Omphalotus olearius*	delta-6-protoilludene	humulene	
A8NE23	Cop6	*Coprinopsis cinerea*	alpha-muurolene	germacrene	[18]
EAU85264	Cop2	*Coprinopsis cinerea*	Germacrene A	germacrene	
EAU85540	Cop4	*Coprinopsis cinerea*	delta-Cadinene	germacrene	
EAU88892	Cop3	*Coprinopsis cinerea*	alpha-Muurolene	germacrene	
EAU89322	Cop1	*Coprinopsis cinerea*	Germacrene A	germacrene	
PubMed	ShSTS12	*Stereum hirsutum*	alpha-cubebene	germacrene	[4]
PubMed	ShSTS10	*Stereum hirsutum*	delta-cadinene	germacrene	
PubMed	ShSTS7	*Stereum hirsutum*	delta-cadinene	germacrene	
PubMed	CPSTS18	*Clitopilus pseudopinsitus*	gamma-cadinene	germacrene	
PubMed	ShSTS5	*Stereum hirsutum*	gamma-cadinene	germacrene	
PubMed	CPSTS2	*Clitopilus pseudopinsitus*	delta-cadinene	germacrene	
PubMed	ShSTS8	*Stereum hirsutum*	alpha-cubebene	germacrene	
PubMed	ShSTS11	*Stereum hirsutum*	alpha-cubebene	germacrene	

PubMed	CPSTS5	*Clitopilus pseudopinsitus*	gamma-muurolene	germacrene	
PubMed	CPSTS3	*Clitopilus pseudopinsitus*	alpha-muurolene	germacrene	
PubMed	CPSTS14	*Clitopilus pseudopinsitus*	beta-elemene	germacrene	
PubMed	CPSTS9	*Clitopilus pseudopinsitus*	ledene	germacrene	
PubMed	CPSTS13	*Clitopilus pseudopinsitus*	ledene	germacrene	
PubMed	CPSTS12	*Clitopilus pseudopinsitus*	beta-elemene	germacrene	
PubMed	CPSTS16	*Clitopilus pseudopinsitus*	aristolene	germacrene	
PubMed	CPSTS8	*Clitopilus pseudopinsitus*	alloaromadendrene	germacrene	
PubMed	CPSTS11	*Clitopilus pseudopinsitus*	9-alloaromadendrene	germacrene	
PubMed	CPSTS1	*Clitopilus pseudopinsitus*	sterprene	humulene	
PubMed	CPSTS6	*Clitopilus pseudopinsitus*	pentalenene	humulene	
PubMed	ShSTS4	*Stereum hirsutum*	hirsutene	humulene	
PubMed	ShSTS16	*Stereum hirsutum*	delta-6-protoilludene	humulene	
PubMed	ShSTS17	*Stereum hirsutum*	delta-6-protoilludene	humulene	
PubMed	ShSTS18	*Stereum hirsutum*	delta-6-protoilludene	humulene	
PubMed	CPSTS17	*Clitopilus pseudopinsitus*	beta-caryophyllene	humulene	
PubMed	ShSTS13	*Stereum hirsutum*	beta-caryophyllene	humulene	
PubMed	CPSTS4	*Clitopilus pseudopinsitus*	6-protoilludene	humulene	
PubMed	CPSTS7	*Clitopilus pseudopinsitus*	alpha-farnesene	acyclic	
PubMed	ShSTS3	*Stereum hirsutum*	beta-farnesene	acyclic	
PubMed	ShSTS15	*Stereum hirsutum*	delta-6-protoilludene	humulene	
JGI ID: 825684	Pilcr_825684	*Piloderma croceum*	gamma-cadinene	germacrene	[19]
JGI ID: 47084	Sphst_47084	*Stereum hirsutum*	Viridiflorol	germacrene	
JGI ID: 659367	Denbi1_659367	*Dendrothele bispora*	delta-6-protoilludene	humulene	
JGI ID: 816208	Denbi1_816208	*Dendrothele bispora*	Viridiflorol	germacrene	
JGI ID: 104215	Galma_104215	*Galerina marginata*	beta-Gurjunene	germacrene	
JGI ID: 454193	Hetan2_454193	*Heterobasidion annosum*	delta-6-protoilludene	humulene	
JGI ID: 138665	Hypsu1_138665	*Hypholoma sublateritium*	delta-6-protoilludene	humulene	
MN146024	Agr1	*Agrocybe aegerita*	delta-cadinene	germacrene	
MN146025	Agr2	*Agrocybe aegerita*	viridiflorene	germacrene	
MN146026	Agr3	*Agrocybe aegerita*	alpha-Muurolene	germacrene	
MN146027	Agr4	*Agrocybe aegerita*	delta-cadinene	germacrene	
MN146028	Agr5	*Agrocybe aegerita*	Viridiflorol	germacrene	
MN146029	Agr6	*Agrocybe aegerita*	delta-6-protoilludene	humulene	
MN146030	Agr7	*Agrocybe aegerita*	delta-6-	humulene	

			protoilludene		
MN146031	Agr8	*Agrocybe aegerita*	gamma-Muurolene	germacrene	
MN146032	Agr9	*Armillaria gallica*	delta-6-protoilludene	humulene	
JGI:77541	SiTPS	*Serendipita indica*	viridiflorene	germacrene	[20]
**Ascomycota**					
A0A516F405	peniA	*Penicillium patulum*	silphiene	humulene	[21]
A0A284RNH4	PRO1	*Armillaria ostoyae*	delta-6-protoilludene	humulene	[22]
PubMed	FgJ09920	*Fusarium graminearum*	koraiol	humulene	[23]
AKE33935.1	CaTPS	*Colletotrichum acutatum*	beta-caryophyllene	humulene	[24]
JGI ID:17536	EC12-PGS	*Daldinia eschscholzii*	alpha-guaiene	germacrene	[25]
JGI ID:24646	EC12-SS	*Daldinia eschscholzii EC12*	alpha-selinene	germacrene	
JGI ID:372695	EC38-CS	*Hypoxylon sp. EC38*	caryophyllene	humulene	
JGI ID:397991	CO27-CS	*Hypoxylon sp. CO27*	alpha-caryophyllene	humulene	
JGI ID:6706	CI4A-CS	*Hypoxylon sp. CI4A*	alpha-caryophyllene	humulene	
JGI ID:70183	EC12-ILS	*Daldinia eschscholzii EC12*	(-)-isoledene	germacrene	
B6H063	prx2	*Penicillium chrysogenum*	(+)-aristolochene	germacrene	[26]
CCT65043	FfSTC3	*Fusarium fujikuroi*	(+)-eremophilene	germacrene	[27]
CCT75704	FfSTC5	*Fusarium fujikuroi*	Guaiadiene	germacrene	
AHY23920	Hyp1	*Hypoxylon sp. E7406B*	nerolidol	acyclic	[28]
AHY23921	Hyp2	*Hypoxylon sp. E7406B*	delta-cadinene	germacrene	
PubMed	Dia1	*Diaporthe sp.*	delta-6-protoilludene	humulene	[29]
PubMed	Tvi09626	*Trichoderma viride*	trichobrasileno	humulene	[30]
CCP20071	Ffsc6	*Fusarium fujikuroi*	koraiol	humulene	[31]
AAQ16575	BcBOT2	*Botrytis cinerea*	presilphiperfolan-8beta-ol	humulene	[32]
Q9UR08	AtAS	*Aspergillus terreus*	(+)-aristolochene	germacrene	[33]
Q03471	Ari1	*Penicillium roqueforti*	(+)-aristolochene	germacrene	[34]
BBK61014.1	TaTC6	*Trichoderma atroviride*	trichobrasileno	humulene	[35]

Table S3. TDTSs selected for heterologous expression.

Gene name	Organism	Division	Identity[#]	Family in SSN
*ni15096-TDTS**	*Nigrospora sp.*	Ascomycota	D1M8S2 (36.46%)	Others
*bb12017-TDTS**	*Beauvaria sp.*	Ascomycota	TvSTS_14 (28.6%)	Others
*an14053-TDTS**	*Arthrinium nigrum*	Ascomycota	FlvE (42.9%)	2
*tv15005-TDTS**	*Trichoderma voglmayrii*	Ascomycota	ShSTS1 (25.94%)	Others
*cs15091-TDTS**	*Cochliobolus sativus*	Ascomycota	D1M8S2 (33.5%)	1
*fs13303-TDTS**	*Fusarium sporotrichioides*	Ascomycota	D1M8S2 (58.5%)	1
*dd13390-TDTS**	*Dicarpella dryina*	Ascomycota	D1M8S2 (31.3%)	1
*gm14063-TDTS-1**	*Glomerella magna*	Ascomycota	LbSTS6 (50.5%)	6
*gm14063-TDTS-2**	*Glomerella magna*	Ascomycota	D1M8S2 (32.5%)	1
*hc11550-TDTS**[=]	*Hapsidospora sp.*	Basdiomycota	PitB (25.90%)	Others
go15085-TDTS	*Ganoderma oregonense*	Basdiomycota	TvSTS_16 (41.2%)	3
to17007-TDTS	*Trichoderma ovalisporum*	Ascomycota	ShSTS1 (26.3%)	Others
ap17081-TDTS	*Arthrinium phaeospermum*	Ascomycota	D1M8S2 (34.1%)	1
hc17010-TDTS	*Hypomyces chrysospermus*	Ascomycota	D1M8S2 (29.9%)	4
fom14004-TDTS	*Fusarium oxysporum*	Ascomycota	D1M8S2 (36.8%)	1
np14056-TDTS[=]	*Neofusicoccum parvum*	Ascomycota	LbSTS6 (40.6%)	6
da15094-TDTS[=]	*Trametes gibbosa*	Basdiomycota	PpSTS29 (35.5%)	9
fc13317-TDTS	*Fusarium culmorum*	Ascomycota	D1M8S2 (38.5%)	7
cc13107-TDTS[=]	*Clitocybe clavipes*	Basdiomycota	AbSTS_07 (30.0%)	2
as13380-TDTS[=]	*Armillaria sinapina*	Basdiomycota	ShSTS1 (44.7%)	5
da15073-TDTS[=]	*Diaporthaceae sp.*	Ascomycota	PitB (29.4%)	Others
ms17109-TDTS[=]	*Mycena sanguinolenta*	Basdiomycota	Q00835 (48.17%)	8
ps13126-TDTS[=]	*Phaeolus schweinitzii*	Basdiomycota	FlvE (29.3%)	Others

* Active TDTSs.

[#] The identity between TDTSs in this work and reported previously.

[=] Synthesized genes.

Table S4. Primers used in this study.

Gene	Restriction Site	Primer sequence 5'-3'	Vector/Size
ap17081-TDTS	*Kpn*I	gcaagctccgAATTCGAGCTCATGGGGGACTGGCAAAC (primer-F)<br>acgagctactacaGATCCCCGCTATTTACGCGTCGAAACCTT (primer-R)	pUARA4<br>1138 bp
gm14063-TDTS-2	*Kpn*I	agctccgAATTCGAGCTCGATGCACATTCAGATGAATGATATT (primer-F)<br>gagctactacaGATCCCCGGTCAAGCCGGACATTCCATAG (primer-R)	pUARA4<br>1198 bp
gm14063-TDTS-1	*Kpn*I	agctccgAATTCGAGCTCGATGCCCATCGAAGCTTCTC (primer-F)<br>gagctactacaGATCCCCGGCTAGACATGCAAGTCAAGTTCT	pUARA4<br>1325 bp
go15085-TDTS	*Kpn*I	aagctccgAATTCGAGCTCGatgtattcattctctctttcccag (primer-F)<br>cgagctactacaGATCCCCGGctaaggtgcggtacagttc (primer-R)	pUARA4<br>1253 bp
ms13281-TDTS	*Kpn*I	aagctccgAATTCGAGCTCATGGTTTCCACAAGTGAAATTTCCA (primer-F)<br>CCGAAGTTTGCGGACGACTAGCGGGGATCtgtagtagctc (primer-R)	pUARA4<br>1064 bp
tv15005-TDTS	*Kpn*I	caagctccgAATTCGAGCTCatggcgagtactttccacag (primer-F)<br>cgagctactacaGATCCCCGttactcagtggctaccatatcctt (primer-R)	pUARA4<br>1153 bp
da15094-TDTS	*Kpn*I	aagctccgAATTCGAGCTCGatggccgaactcgattctag (primer-F)<br>gagctactacaGATCCCCGGtcacatttgcgcaggcg	pUARA4<br>1076 bp
dd13390-TDTS	*Kpn*I	caagctccgAATTCGAGCTCATGGGTTGCCACGAAAATGA (primer-F)<br>cgagctactacaGATCCCCGTTAGTTGCTGAAAACCCGTT (primer-R)	pUARA4<br>1300 bp
fs13303-TDTS	*Kpn*I	agctccgAATTCGAGCTCGATGATCGCTGCACCGAG (primer-F)<br>gagctactacaGATCCCCGGTTAGTTCCTCTGGACAATTTCCTTT	pUARA4<br>1278 bp
fom14004-TDTS	*Kpn*I	caagctccgAATTCGAGCTCatgtttatggtcggaagcca (primer-F)<br>cgagctactacaGATCCCCGctaaggccccagcgc (primer-R)	pUARA4<br>1152 bp
au11219-TDTS	*Kpn*I	cgAATTCGAGCTCGGTACCATGCTTCAACCCAGTTCAGTT (primer-F)<br>GACGGTGATCTCGTTTGCTAACGGGGATCtgtagtagctc (primer-R)	pUARA4<br>1321 bp
pm17515-TDTS	*Kpn*I	aagctccgAATTCGAGCTCATGGACCGTATTACTCCCAAA (primer-F)<br>gagctactacaGATCCCCGCTATGATCCAAACGACCACAC (primer-R)	pUARA4<br>1252 bp
di11573-TDTS	*Kpn*I	aagctccgAATTCGAGCTCATGTTGCGCAATTATGTGCGT (primer-F)<br>gagctactacaGATCCCCGTCAGACAACAAAAGCTGCCTG (primer-R)	pUARA4<br>1352 bp
bb12017-	*Kpn*I	agctccgAATTCGAGCTCGATGACTCCAGGC	pUARA4

TDTS		ACGGA (primer-F)<br>gagctactacaGATCCCCGGTTATTGGATAGCTATTCCGATTTCCTTC (primer-R)	975 bp
ph10046-TDTS	*Kpn*I	aagctccgAATTCGAGCTCATGTCCCATTCACGCCACGAC (primer-F)<br>GTTACTGCCGTAGAGGTTTGACGGGGATCtgtagtagctc (primer-R)	pUARA4<br>1274 bp
hc17010-TDTS	*Kpn*I	GcaagctccgAATTCGAGCTCATGGAGCCCAAGGTCTTC (primer-F)<br>acgagctactacaGATCCCCGTCACCGATTTTGCCATTCATC (primer-R)	pUARA4<br>1164 bp
hc11500-TDTS	*Kpn*I	agctccgAATTCGAGCTCATGGTATGAACCGTTTGAAAAAGC (primer-F)<br>cgagctactacaGATCCCCGtcagGCTCTGCAAGTCTGCA (primer-R)	pUARA4<br>1251 bp
cs15091-TDTS	*Kpn*I	aagctccgAATTCGAGCTCATGGGACATTCTGCCAAAGAC (primer-F)<br>cgagctactacaGATCCCCGCTAGCCTGCAAACACTTCCT (primer-R)	pUARA4<br>1140 bp
an14053-TDTS	*Kpn*I	GcaagctccgAATTCGAGCTCATGACTGCTGCAGAGTTC (primer-F)<br>acgagctactacaGATCCCCGTCAGCGAACCAGAACCC (primer-R)	pUARA4<br>1161 bp
np14056-TDTS	*Kpn*I	GcaagctccgAATTCGAGCTCATGCATCTCGCGATAGAACA (primer-F)<br>cgagctactacaGATCCCCGCTAAAGATGACTTTGCGTCTCC (primer-R)	pUARA4<br>1354 bp
fc13317-TDTS	*Kpn*I	AGCTCCGAATTCGAGCTCATGGGCATTTGGCTATCA (primer-F)<br>ACGAGCTACTACAGATCCCCGTTATGTATCGATGCTCTGAGCT (primer-R)	pUARA4<br>1247 bp
ni15096-TDTS	*Kpn*I	AGCTCCGAATTCGAGCTCATGGTCTGTAGCATTTTCTGTTCAACAGTA (primer-F)<br>ACGAGCTACTACAGATCCCCGTTAGGAAGTCTTGGCCACCTCCTT (primer-R)	pUARA4<br>1578 bp

Table S5. Plasmids and transformants constructed in this study.

Plasmid	Gene to be expressed	Transformant
pUARA4-*ap17081-TDTS*	*ap17081-TDTS*	AO-*ap17081-TDTS*
pUARA4-*gm14063-TDTS-2*	*gm14063-TDTS-2*	AO-*gm14063-TDTS-2*
pUARA4-*gm14063-TDTS-1*	*gm14063-TDTS-1*	AO-*gm14063-TDTS-1*
pUARA4-*go15085-TDTS*	*go15085-TDTS*	AO-*go15085-TDTS*
pUARA4-*ms13281-TDTS*	*ms13281-TDTS*	AO-*ms13281-TDTS*
pUARA4-*tv15005-TDTS*	*tv15005-TDTS*	AO-*tv15005-TDTS*
pUARA4-*da15094-TDTS*	*da15094-TDTS*	AO-*da15094-TDTS*
pUARA4-*dd13390-TDTS*	*dd13390-TDTS*	AO-*dd13390-TDTS*
pUARA4-*fs13303-TDTS*	*fs13303-TDTS*	AO-*fs13303-TDTS*
pUARA4-*fom14004-TDTS*	*fom14004-TDTS*	AO-*fom14004-TDTS*
pUARA4-*au11219-TDTS*	*au11219-TDTS*	AO-*au11219-TDTS*
pUARA4-*pm17515-TDTS*	*pm17515-TDTS*	AO-*pm17515-TDTS*
pUARA4-*di11573-TDTS*	*di11573-TDTS*	AO-*di11573-TDTS*
pUARA4-*bb12017-TDTS*	*bb12017-TDTS*	AO-*bb12017-TDTS*
pUARA4-*ph10046-TDTS*	*ph10046-TDTS*	AO-*ph10046-TDTS*
pUARA4-*hc17010-TDTS*	*hc17010-TDTS*	AO-*hc17010-TDTS*
pUARA4-*hc11500-TDTS*	*hc11500-TDTS*	AO-*hc11500-TDTS*
pUARA4-*cs15091-TDTS*	*cs15091-TDTS*	AO-*cs15091-TDTS*
pUARA4-*an14053-TDTS*	*an14053-TDTS*	AO-*an14053-TDTS*
pUARA4-*np14056-TDTS*	*np14056-TDTS*	AO-*np14056-TDTS*
pUARA4-*fc13317-TDTS*	*fc13317-TDTS*	AO-*fc13317-TDTS*
pUARA4-*ni15096-TDTS*	*ni15096-TDTS*	AO-*ni15096-TDTS*

Reference

1. Zhang, T.; Feng, J.; He, W.; Rong, X.; Lv, H.; Li, J.; Li, X.; Wang, H.; Wang, L.; Zhang, L.; et al. Genomic and transcriptomic approaches povide a pedictive framework for sesquiterpenes bosynthesis in *Desarmillaria tabescens* CPCC 401429. *J Fungi (Basel)* **2023**, *9*, 481, doi:10.3390/jof9040481.
2. Lopez-Gallego, F.; Agger, S.A.; Abate-Pella, D.; Distefano, M.D.; Schmidt-Dannert, C. Sesquiterpene synthases Cop4 and Cop6 from *Coprinus cinereus*: catalytic promiscuity and cyclization of farnesyl pyrophosphate geometric isomers. *Chembiochem* **2010**, *11*, 1093–1106, doi:10.1002/cbic.200900671.
3. Wawrzyn, G.T.; Quin, M.B.; Choudhary, S.; Lopez-Gallego, F.; Schmidt-Dannert, C. Draft genome of *Omphalotus olearius* provides a predictive framework for sesquiterpenoid natural product biosynthesis in Basidiomycota. *Chem Biol* **2012**, *19*, 772–783, doi:10.1016/j.chembiol.2012.05.012.
4. Shota, N.; Chengwei, L.; Jumpei, N.; Takuto, K.; Kaho, S.; Yoshiro, S.; Atsushi, M.; Taro, O.; Claudia, S.; Maruyama, J.; et al. Ascomycete *Aspergillus oryzae* is an efficient expression host for production of basidiomycete terpenes by using genomic DNA sequences. *Appl Environ Microbiol* **2019**, *85(15)*, e00409–00419, doi:10.1128/AEM.
5. Masunaga, N.; Kitaoka, T.; Ichinose, H. Biocatalyst collection and heterologous expression of sesquiterpene synthases from basidiomycetous fungi: Discovery of a novel sesquiterpene hydrocarbon. *Microb Biotechnol* **2022**, *16*, 632–644, doi:10.1111/1751-7915.14204.
6. Bahadoor, A.; Schneiderman, D.; Gemmill, L.; Bosnich, W.; Blackwell, B.; Melanson, J.E.; McRae, G.; Harris, L.J. Hydroxylation of longiborneol by a Clm2-encoded CYP450 monooxygenase to produce culmorin in *Fusarium graminearum*. *J Nat Prod* **2016**, *79*, 81–88, doi:10.1021/acs.jnatprod.5b00676.
7. Yee, D.A.; Kakule, T.B.; Cheng, W.; Chen, M.; Chong, C.T.Y.; Hai, Y.; Hang, L.F.; Hung, Y.S.; Liu, N.; Ohashi, M.; et al. Genome mining of alkaloidal terpenoids from a hybrid terpene and nonribosomal peptide biosynthetic pathway. *J Am Chem Soc* **2020**, *142*, 710–714, doi:10.1021/jacs.9b13046.
8. Nosenko, T.; Zimmer, I.; Ghirardo, A.; Kollner, T.G.; Weber, B.; Polle, A.; Rosenkranz, M.; Schnitzler, J.P. Predicting functions of putative fungal sesquiterpene synthase genes based on multiomics data analysis. *Fungal Genet Biol* **2023**, *165*, 103779, doi:10.1016/j.fgb.2023.103779.
9. Proctor, R.H.; McCormick, S.P.; Kim, H.S.; Cardoza, R.E.; Stanley, A.M.; Lindo, L.; Kelly, A.; Brown, D.W.; Lee, T.; Vaughan, M.M.; et al. Evolution of structural diversity of trichothecenes, a family of toxins produced by plant pathogenic and entomopathogenic fungi. *PLoS Pathog* **2018**, *14*, e1006946, doi:10.1371/journal.ppat.1006946.
10. Ichinose, H.; Kitaoka, T. Insight into metabolic diversity of the brown-rot basidiomycete *Postia placenta* responsible for sesquiterpene biosynthesis: semi-comprehensive screening of cytochrome P450 monooxygenase involved in protoilludene metabolism. *Microb Biotechnol* **2018**, *11*, 952–965,

doi:10.1111/1751-7915.13304.
11. Li, R.; Yao, B.; Zeng, H. Identification and characterization of a nerol synthase in fungi. *J Agric Food Chem* **2023**, *72*, 416–423, doi:10.1021/acs.jafc.3c07573.
12. Ichinose, H.; Ukeba, S.; Kitaoka, T. Latent potentials of the white-rot basidiomycete Phanerochaete chrysosporium responsible for sesquiterpene metabolism: CYP5158A1 and CYP5144C8 decorate (E)-alpha-bisabolene. *Enzyme Microb Technol* **2022**, *158*, 110037, doi:10.1016/j.enzmictec.2022.110037.
13. Al-Salihi, S.A.A.; Dao, T.T.; Williams, K.; Bailey, A.M.; Foster, G.D. The biogenetic origin of the biologically active naematolin of *Hypholoma* species involves an unusual sesquiterpene synthase. *Mol Biotechnol* **2019**, *61*, 754–762, doi:10.1007/s12033-019-00199-x.
14. Burkhardt, I.; Kreuzenbeck, N.B.; Beemelmanns, C.; Dickschat, J.S. Mechanistic characterization of three sesquiterpene synthases from the termite-associated fungus *Termitomyces*. *Org Biomol Chem* **2019**, *17*, 3348–3355, doi:10.1039/c8ob02744g.
15. Lackner, G.; Peters, E.E.; Helfrich, E.J.; Piel, J. Insights into the lifestyle of uncultured bacterial natural product factories associated with marine sponges. *Proc Natl Acad Sci U S A* **2017**, *114*, E347–E356, doi:10.1073/pnas.1616234114.
16. Mischko, W.; Hirte, M.; Fuchs, M.; Mehlmer, N.; Bruck, T.B. Identification of sesquiterpene synthases from the Basidiomycota *Coniophora puteana* for the efficient and highly selective beta-copaene and cubebol production in *E. coli*. *Microb Cell Fact* **2018**, *17*, 164, doi:10.1186/s12934-018-1010-z.
17. Yap, H.Y.; Muria-Gonzalez, M.J.; Kong, B.H.; Stubbs, K.A.; Tan, C.S.; Ng, S.T.; Tan, N.H.; Solomon, P.S.; Fung, S.Y.; Chooi, Y.H. Heterologous expression of cytotoxic sesquiterpenoids from the medicinal mushroom *Lignosus rhinocerotis* in yeast. *Microb Cell Fact* **2017**, *16*, 103, doi:10.1186/s12934-017-0713-x.
18. Agger, S.; Lopez-Gallego, F.; Schmidt-Dannert, C. Diversity of sesquiterpene synthases in the basidiomycete *Coprinus cinereus*. *Molecular Microbiology* **2009**, *72*, 1181–1195, doi:10.1111/j.1365-2958.2009.06717.x.
19. Zhang, C.; Chen, X.; Orban, A.; Shukal, S.; Birk, F.; Too, H.P.; Ruhl, M. *Agrocybe aegerita* serves as a gateway for identifying sesquiterpene biosynthetic enzymes in higher fungi. *ACS Chem Biol* **2020**, *15*, 1268–1277, doi:10.1021/acschembio.0c00155.
20. Ntana, F.; Bhat, W.W.; Johnson, S.R.; Jorgensen, H.J.L.; Collinge, D.B.; Jensen, B.; Hamberger, B. A sesquiterpene synthase from the endophytic fungus *Serendipita indica* catalyzes formation of viridiflorol. *Biomolecules* **2021**, *11*, 898, doi:10.3390/biom11060898.
21. Zeng, H.; Yin, G.; Wei, Q.; Li, D.; Wang, Y.; Hu, Y.; Hu, C.; Zou, Y. Unprecedented [5.5.5.6]dioxafenestrane ring construction in fungal insecticidal sesquiterpene biosynthesis. *Angew Chem Int Ed Engl* **2019**, *58*, 6569–6573, doi:10.1002/anie.201813722.

22. Li, Z.; Wang, Y.; Jiang, B.; Li, W.; Zheng, L.; Yang, X.; Bao, Y.; Sun, L.; Huang, Y.; Li, Y. Structure, cytotoxic activity and mechanism of protoilludane sesquiterpene aryl esters from the mycelium of *Armillaria mellea. J Ethnopharmacol* **2016**, *184*, 119–127, doi:10.1016/j.jep.2016.02.044.
23. Yuan, Y.; Litzenburger, M.; Cheng, S.; Bian, G.; Hu, B.; Yan, P.; Cai, Y.; Deng, Z.; Bernhardt, R.; Liu, T. Sesquiterpenoids produced by combining two sesquiterpene cyclases with promiscuous myxobacterial CYP260B1. *Chembiochem* **2019**, *20*, 677–682, doi:10.1002/cbic.201800670.
24. Buchvaldt Amby, D.; Manczak, T.; Petersen, M.A.; Sundelin, T.; Weitzel, C.; Grajewski, M.; Simonsen, H.T.; Jensen, B. Role of the *Colletotrichum acutatum* sesquiterpene synthase CaTPS in the biosynthesis of sesquiterpenoids. *Microbiology (Reading)* **2016**, *162*, 1773–1783, doi:10.1099/mic.0.000361.
25. Wu, W.; Tran, W.; Taatjes, C.A.; Alonso-Gutierrez, J.; Lee, T.S.; Gladden, J.M. Rapid discovery and functional characterization of terpene synthases from four endophytic *Xylariaceae*. *PLoS One* **2016**, *11*, e0146983, doi:10.1371/journal.pone.0146983.
26. Hidalgo, P.I.; Ullan, R.V.; Albillos, S.M.; Montero, O.; Fernandez-Bodega, M.A.; Garcia-Estrada, C.; Fernandez-Aguado, M.; Martin, J.F. Molecular characterization of the PR-toxin gene cluster in *Penicillium roqueforti* and *Penicillium chrysogenum*: cross talk of secondary metabolite pathways. *Fungal Genet Biol* **2014**, *62*, 11–24, doi:10.1016/j.fgb.2013.10.009.
27. Burkhardt., I.; Siemon., T.; Henrot., M.; Studt., L.; Rçsler., S.; Tudzynski., B.; Christmann., M.; Dickschat, J.S. Mechanistic characterisation of two sesquiterpene cyclases from the plant pathogenic fungus *Fusarium fujikuroi*. *Angew. Chem. Int. Ed* **2016**, *55*, 8748–8751, doi: 10.1002/anie.201603782.
28. Shaw, J.J.; Berbasova, T.; Sasaki, T.; Jefferson-George, K.; Spakowicz, D.J.; Dunican, B.F.; Portero, C.E.; Narvaez-Trujillo, A.; Strobel, S.A. Identification of a fungal 1,8-cineole synthase from *Hypoxylon* sp. with specificity determinants in common with the plant synthases. *J Biol Chem* **2015**, *290*, 8511–8526, doi:10.1074/jbc.M114.636159.
29. de Sena Filho, J.G.; Quin, M.B.; Spakowicz, D.J.; Shaw, J.J.; Kucera, K.; Dunican, B.; Strobel, S.A.; Schmidt-Dannert, C. Genome of *Diaporthe* sp. provides insights into the potential inter-phylum transfer of a fungal sesquiterpenoid biosynthetic pathway. *Fungal Biol* **2016**, *120*, 1050–1063, doi:10.1016/j.funbio.2016.04.001.
30. Sun, X.; Cai, Y.S.; Yuan, Y.; Bian, G.; Ye, Z.; Deng, Z.; Liu, T. Genome mining in *Trichoderma viride* J1-030: discovery and identification of novel sesquiterpene synthase and its products. *Beilstein J Org Chem* **2019**, *15*, 2052–2058, doi:10.3762/bjoc.15.202.
31. Brock, N.L.; Huss, K.; Tudzynski, B.; Dickschat, J.S. Genetic dissection of sesquiterpene biosynthesis by *Fusarium fujikuroi*. *Chembiochem* **2013**, *14*, 311–315, doi:10.1002/cbic.201200695.
32. Moraga, J.; Dalmais, B.; Izquierdo-Bueno, I.; Aleu, J.; Hanson, J.R.;

Hernandez-Galan, R.; Viaud, M.; Collado, I.G. Genetic and molecular basis of botrydial biosynthesis: connecting cytochrome P450-encoding genes to biosynthetic intermediates. *ACS Chem Biol* **2016**, *11*, 2838–2846, doi:10.1021/acschembio.6b00581.
33. Proctor, R.H.; Hohn, T.M. Aristolochene synthase. Isolation, characterization, and bacterial expression of a sesquiterpenoid biosynthetic gene (Ari1) from *Penicillium roqueforti*. *Journal of Biological Chemistry* **1993**, *268*, 4543–4548, doi:10.1016/s0021-9258(18)53644-9.
34. Cane, D.E.; Kang, I. Aristolochene synthase: purification, molecular cloning, high-level expression in *Escherichia coli*, and characterization of the *Aspergillus terreus* cyclase. *Arch Biochem Biophys* **2000**, *376*, 354–364, doi:10.1006/abbi.2000.1734.
35. Murai, K.; Lauterbach, L.; Teramoto, K.; Quan, Z.; Barra, L.; Yamamoto, T.; Nonaka, K.; Shiomi, K.; Nishiyama, M.; Kuzuyama, T.; et al. An unusual skeletal rearrangement in the biosynthesis of the sesquiterpene trichobrasilenol from *Trichoderma*. *Angew Chem Int Ed Engl* **2019**, *58*, 15046–15050, doi:10.1002/anie.201907964.
